# The plasmid‐borne quinolone resistance protein QnrB, a novel DnaA‐binding protein, increases the bacterial mutation rate by triggering DNA replication stress

**DOI:** 10.1111/mmi.14235

**Published:** 2019-03-27

**Authors:** Xiaojing Li, Yujiao Zhang, Xintong Zhou, Xinling Hu, Yixuan Zhou, Di Liu, Anthony Maxwell, Kaixia Mi

**Affiliations:** ^1^ CAS Key Laboratory of Pathogenic Microbiology and Immunology, Institute of Microbiology Chinese Academy of Sciences Beijing 100101 China; ^2^ Savaid Medical School University of Chinese Academy of Sciences Beijing 101408 China; ^3^ Department of Biological Chemistry John Innes Centre Norwich Research Park, Norwich NR4 7UH UK

## Abstract

Bacterial antibiotic resistance, a global health threat, is caused by plasmid transfer or genetic mutations. Quinolones are important antibiotics, partially because they are fully synthetic and resistance genes are unlikely to exist in nature; nonetheless, quinolone resistance proteins have been identified. The mechanism by which plasmid‐borne quinolone resistance proteins promotes the selection of quinolone‐resistant mutants is unclear. Here, we show that QnrB increases the bacterial mutation rate. Transcriptomic and genome sequencing analyses showed that QnrB promoted gene abundance near the origin of replication (*oriC*). In addition, the QnrB expression level correlated with the replication origin to terminus (*oriC*/*ter*) ratio, indicating QnrB‐induced DNA replication stress. Our results also show that QnrB is a DnaA‐binding protein that may act as an activator of DNA replication initiation. Interaction of QnrB with DnaA promoted the formation of the DnaA‐*oriC* open complex, which leads to DNA replication over‐initiation. Our data indicate that plasmid‐borne QnrB increases bacterial mutation rates and that genetic changes can alleviate the fitness cost imposed by transmitted plasmids. Derivative mutations may impair antibiotic efficacy and threaten the value of antibiotic treatments. Enhanced understanding of how bacteria adapt to the antibiotic environment will lead to new therapeutic strategies for antibiotic‐resistant infections.

## Introduction

Bacterial antibiotic resistance is a significant threat to the prevention of infectious diseases (Davies, 1994; Berendonk *et al.*, 2015; Kling *et al.*, 2015; Morehead and Scarbrough, 2018) that generally results from plasmid transfer or genetic mutations (Toprak *et al.*, 2011; San Millan, 2018). High mutation rates can occur in response to environmental factors (Bell and Gonzalez, 2011; Jolivet‐Gougeon *et al.*, 2011), including exposure to antibiotics, and increasing mutation rates have certainly contributed to the emergence of antibiotic resistance in the clinical setting (Kohanski *et al.*, 2010; Krasovec *et al.*, 2014). Plasmids drive the horizontal transfer of antibiotic resistance genes, and compensatory mutations can alleviate the fitness cost imposed by the transmitted plasmid (Gama *et al.*, 2018; San Millan, 2018). Furthermore, interactions between plasmids and the bacterial chromosome impact the spread of antibiotic resistance (Gama *et al.*, 2018). Understanding the mechanisms underlying these processes will provide insights into how bacteria adapt to an antibiotic environment and will aid the optimization of antimicrobial strategies.

Quinolones, used as anti‐infection agents, are fully synthetic in origin (Mitscher, 2005). For a long time, resistance to quinolones was thought to be caused by mutation of their target genes (encoding DNA gyrase and DNA topoisomerase IV) and/or changes in cell wall permeability. It was assumed that no quinolone resistance genes existed naturally. It is now known that quinolone resistance (Qnr) proteins cause low‐level quinolone resistance and facilitate the selection of resistant mutants (Redgrave *et al.*, 2014). Since the first plasmid encoding a Qnr protein was discovered (Martinez‐Martinez *et al.*, 1998; Tran and Jacoby, 2002), a large number of plasmid‐borne Qnr proteins have been described, and bacterial species harboring these proteins are ubiquitous (Hooper and Jacoby, 2015; Ruiz, 2018). The *mfpA* gene, which affects fluoroquinolone susceptibility (Montero *et al.*, 2001), was the first chromosomal *qnr* gene to be identified. Subsequently, Qnr chromosomal homologs have been identified in many organisms (Jacoby and Hooper, 2013). MfpA has a DNA‐mimicking structure and is predicted to interact with DNA gyrase and protect it against drug‐induced damage (Hegde *et al.*, 2005). Indeed, previous studies have mainly focused on the interaction of Qnr proteins with DNA topoisomerases (Shah and Heddle, 2014; Rodriguez‐Martinez *et al.*, 2016), and the native functions of Qnr proteins are generally thought to be related to their effects on DNA gyrase; however, no direct experiments support this speculation. Several studies have shown that Qnr proteins facilitate the selection of antibiotic‐resistant mutants (Redgrave *et al.*, 2014; Vinue *et al.*, 2015). However, the molecular mechanisms by which Qnr proteins facilitate genetic variations that affect susceptibility to the quinolone ciprofloxacin (CIP) are unclear.

In this study, we examined the effect of QnrB on the mutation rate of the *Escherichia coli* BW25113 strain (Baba *et al.*, 2006) and the *Klebsiella pneumoniae* KP48 clinical strain (Jiang *et al.*, 2010). Luria and Delbruck fluctuation analyses revealed that QnrB increases the bacterial mutation rate. Furthermore, transcriptomic and whole genome sequencing analyses showed that QnrB upregulates gene expression and increases the number of gene copies near the origin replication (*oriC*) in both *E. coli* and *K. pneumoniae*. In addition to an increase in *qnrB* mRNA, marker frequency analysis showed an increase in replication origin to terminus (*oriC*/ter) ratio, indicating DNA replication stress, in both *E. coli *and *K. pneumoniae*. Bacterial two‐hybrid and *in vitro* pull‐down assays showed that QnrB interacts with the DNA replication initiator DnaA. In addition, microscale thermophoresis (MST) and *oriC* unwinding assays showed that QnrB increases the affinity of DnaA for single‐stranded (ss) *oriC* and promotes the formation of the DnaA‐*oriC* open complex, which leads to DNA replication stress and an accumulation of mutations, including those promoting quinolone resistance. Overall, our results show that plasmid‐borne QnrB promotes bacterial mutation and the selection of quinolone‐resistant mutants.

## Results

### QnrB increases the bacterial mutation rate

**Figure 1:**
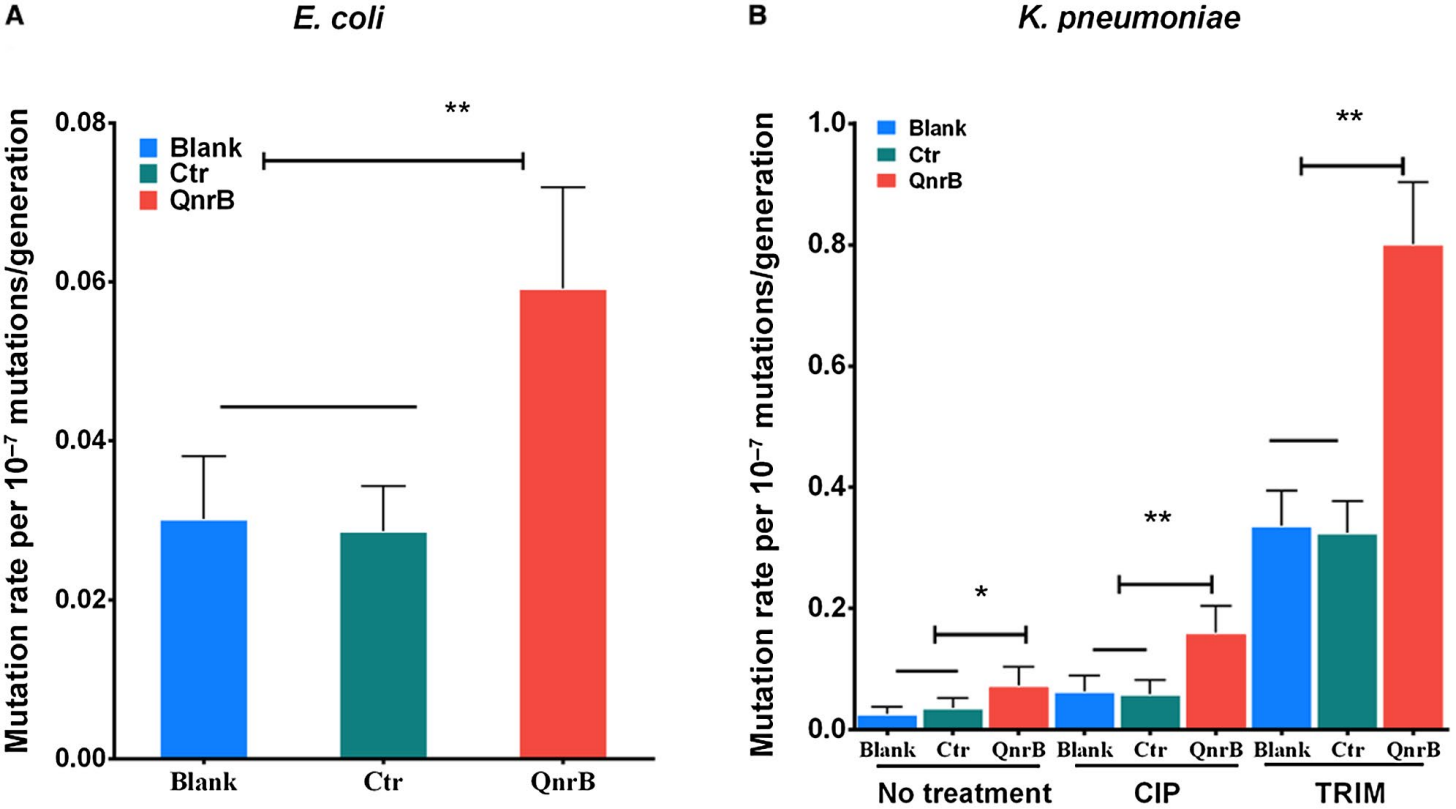
QnrB increases the bacterial mutation rate. A. Luria and Delbruck fluctuation analysis of the mutation rates (mean ± 95% CI, see also Table S1) in *E. coli* BW25113 strains carrying pQE80L (pQE80L/BW25113; Blank), pQE‐*msmeg_2415 *(pQE‐*msmeg_2415*/BW25113; Ctr) or pQE‐*qnrB* (QnrB). B. Luria and Delbruck fluctuation analysis of the mutation rates (mean ± 95% CI, see also Table S1) in *K. pneumoniae *strains carrying pACYC/KP49 (Blank), pACYC‐*msmeg_2415*/KP48 (negative control, Ctr) or pACYC‐*qnrB*/KP49 (QnrB). The cells were treated with or without CIP (96 mg/l) or TRIM (0.5 mg/l) as indicated. A, B. A significant difference was observed in the mutation rate between the strain expressing QnrB and the strain lacking QnrB (mean ± 95% CI, two‐sided Student’s *t*‐test, ^**^ *p* < 0.01, ^*^ *p* < 0.1). The data shown are representative of five independent experiments. CIP, ciprofloxacin; TRIM, trimethoprim; Ctr, negative control; Blank, blank control. [Colour figure can be viewed at wileyonlinelibrary.com]

Previous studies showed that, in the presence of *qnr* genes, a variety of mutations are selected to enhance CIP resistance (Cesaro *et al.*, 2008; Vinue *et al.*, 2015); therefore, we hypothesized that Qnr proteins can increase the bacterial mutation rate. To test this hypothesis, we amplified the *qnrB *gene from the *K. pneumoniae *multidrug‐resistant pKP048 plasmid (Jiang *et al.*, 2010), which carries several resistance‐related genes, including *qnrB*, *bla*_kpc‐2_, *bla*_DHA‐1_ and *armA *(Jiang *et al.*, 2010), and constructed *E. coli* BW25113 strains harboring pQE‐*qnrB* (expressing the His_6_‐QnrB protein) or pQE80L (empty vector) as a control. A Luria and Delbruck fluctuation analysis (Luria and Delbruck, 1943; Sarkar *et al.*, 1992) was used to examine mutation of the *rpoB* gene, related to rifampicin resistance, in the two cell types, and the mutation rate was calculated using the MSS Maximum Likelihood Estimation method (Hall *et al.*, 2009). Expression of QnrB in *E. coli* resulted in a twofold increase in the mutation rate (Fig. 1A and Table S1). Previous studies showed that overexpression of recombinant proteins may trigger bacterial stress, leading to an increased mutation rate (Hoffmann and Rinas, 2004; Krasovec *et al.*, 2014). However, overexpression of MSMEG_2415 (pQE‐*msmeg_2415*) (Li *et al.*, 2014), a hemerythrin‐like protein from *Mycobacterium smegmatis*, did not affect the mutation rate in *E. coli* BW25113 cells (Fig. 1A and Table S1), indicating that the increased mutation rate was caused by QnrB specifically, rather than general stress related to overexpression of a recombinant protein.

Next, we examined the effect of QnrB on the mutation rate of a clinical *K. pneumoniae* strain KP48 carrying the conjugated pKP048 plasmid. To avoid interference by other resistance‐related genes, the native pKP048 plasmid in the strain was eliminated by treatment with sodium dodecyl sulfate at 42°C (El‐Mansi *et al.*, 2000). The KP49 was a cured strain that had lost its native pKP048 plasmid. Subsequently, the *qnrB* gene from pKP048 was amplified and cloned into the pACYC vector, which is useful for expressing recombinant proteins in *K. pneumoniae *(Xu *et al.*, 2017), and strains containing the resulting construct (pACYC‐*qnrB*/KP49), or the empty vector (pACYC/KP49) as a control, were generated. QnrB expression was detected using an anti‐His antibody (Fig. S1). The mutation rate of the pACYC‐*qnrB*/KP49 strain was more than twice that of the pACYC/KP49 strain (Fig. 1B and Table S1). Furthermore, as seen in *E. coli*, overexpression of MSMEG_2415 did not affect the mutation rate of *K. pneumoniae* cells (Fig. 1B and Table S1), indicating that the observed increase was attributed to QnrB specifically.

Subsequently, we examined the effect of QnrB on the mutation rate in *K. pneumoniae* cells treated with the quinolone CIP. Previous studies showed that quinolones can increase the mutation rate, at least in part, by inducing SOS responses (Ysern *et al.*, 1990; Cirz and Romesberg, 2006). CIP treatment increased the mutation rate of all test strains (pACYC/KP49, pACYC‐*msmeg_2415*/KP49 and pACYC‐*qnrB*/KP49) by approximately twofold (Fig. 1B and Table S1). Based on the increase in the overall mutation rate of all test strains, the mutation rate of pACYC‐*qnrB*/KP49 was twofold higher than that of the strains lacking QnrB (Fig. 1B and Table S1). As a possible explanation for this result, QnrB is a DNA mimic protein that affects the action of gyrase and blocks progression of the DNA replication fork, resulting in increased mutation rates.

Next, we examined the effect of another antibiotic, trimethoprim (TRIM), on mutation rates in the pACYC/KP49, pACYC‐*msmeg_2415*/KP49 and pACYC‐*qnrB*/KP49 strains. TRIM causes thymineless death and an increased mutation rate due to an enhanced SOS response (Fonville *et al.*, 2010), irrespective of gyrase function. TRIM treatment increased the mutation rate of all three test strains by approximately 8‐10‐fold (Fig. 1B and Table S1). Based on the increase in the overall mutation rate of all test strains, the mutation rate of pACYC‐*qnrB*/KP49 was twofold higher than that of the strains lacking QnrB (Fig. 1B and Table S1). Overall, these results show that QnrB expression increases the bacterial mutation rate in a manner that may be independent of gyrase inhibition.

### QnrB expression is correlated with upregulation of genes proximal to oriC

**Figure 2:**
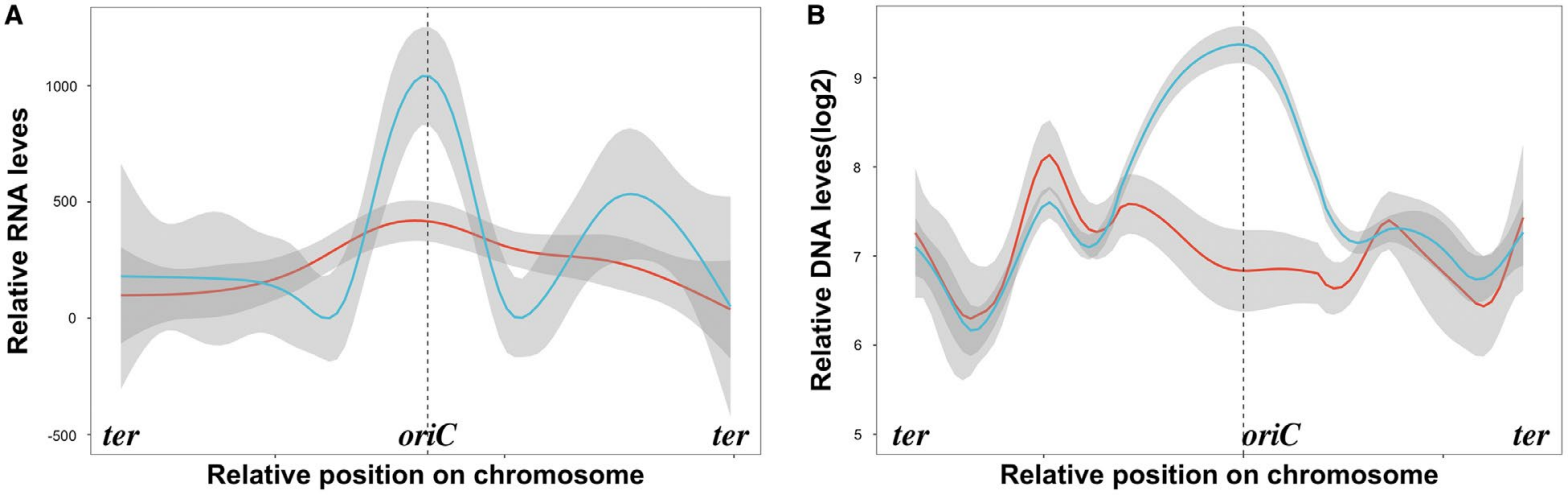
QnrB expression is correlated with upregulation of *oriC*‐proximal genes. A. Global transcriptional responses of the *K. pneumoniae* KP48 and KP49 strains. Gene expression (log2) was determined by RNA sequencing after treatment of the cells with CIP for 30 min. FPKM (Fragments Per Kilobase of transcript per Million mapped reads) values for expressed genes (with a log2‐fold‐change > 2 or <−2) are plotted on the chromosome. The error ranges are illustrated by the shaded regions. The red and blue lines indicate KP49 lacking QnrB and KP48 expressing QnrB strains respectively. B. Genome‐wide marker frequency analysis using whole genome sequencing. The medians were read on a sliding window of 293 genes. The shaded regions indicate the coverage deviation from the median of these reads within the window. The red and blue lines indicate the changes in the gene copy numbers in CIP‐treated KP48 and KP49 cells respectively. FPKM, Fragments Per Kilobase of transcripts sequence per Millions base pairs sequenced. CIP, ciprofloxacin. [Colour figure can be viewed at wileyonlinelibrary.com]

To determine the molecular mechanism by which QnrB increases bacterial mutation rates, we compared the global transcriptional responses of *K. pneumoniae* strains lacking or expressing *qnrB*. A previous study showed that CIP can induce replication stress and upregulate genes located close to *oriC *(Slager *et al.*, 2014). Consistent with this finding, an increased transcript peak near *oriC* was observed in KP48 and KP49 cells treated with CIP, although the increase was more pronounced for the strain expressing QnrB (Fig. 2A). We hypothesized that, if QnrB overstimulates DNA replication, the copy numbers of genes near *oriC* would also increase. To test this hypothesis, we performed genome sequencing of KP48 and KP49 strains and found that QnrB expression was associated with a higher copy number of *oriC*‐proximal genes (Fig. 2B). Taken together, these results show that QnrB promotes DNA replication stress and leads to upregulation of genes near *oriC*.

To confirm that QnrB upregulates *oriC*‐proximal genes, we compared the copy numbers and expression levels of genes near *oriC* in *E. coli* strains with and without QnrB expression. Genomic DNA and RNA were purified from cultures of *E. coli* BW25113 cells harboring a plasmid containing the *qnrB* gene under control of the *araB* promoter (Table S2). In this strain, arabinose and glucose induced and inhibited *qnrB* expression respectively. As seen in *K. pneumoniae*, QnrB expression upregulated *oriC*‐proximal gene expression in this *E. coli* strain (Fig. S2), suggesting that it promotes DNA replication stress.

### QnrB induces DNA replication stress

**Figure 3:**
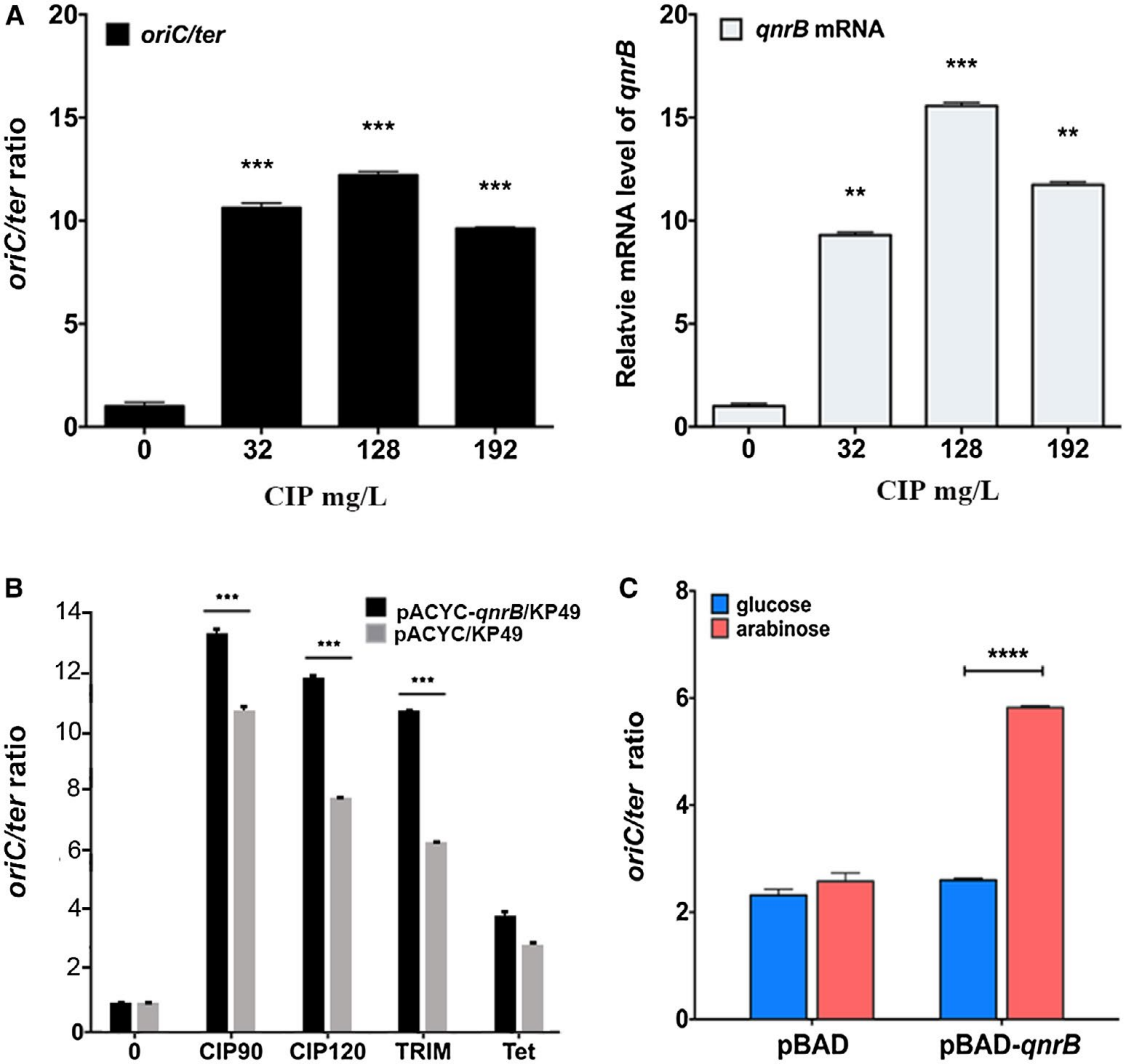
QnrB increases DNA replication stress. A. CIP induces replication stress (*oriC/ter* ratio; left panel) and *qnrB* mRNA expression (right panel) in a concentration‐dependent manner. The *K. pneumoniae* KP48 strain harboring a plasmid expressing QnrB was treated with or without CIP (0, 32, 128 or 192 mg/l) for 90 min. The *oriC/ter* ratio and *qnrB* mRNA level were measured using real‐time quantitative PCR. Data are shown as the mean ± SD of three replicates. A significant difference in the *oriC*/ter ratio was observed between the strain with CIP and the strain without CIP (two‐sided Student’s *t*‐test, ^**^ *p* < 0.01, ^***^ *p* < 0.001). The data shown are representative of three independent experiments. B. TRIM induces replication stress. The *K. pneumoniae* KP48 and KP49strains were treated with or without CIP (128 mg/l), TRIM (0.7 mg/l) or tetracycline (Tet; 1 mg/l) for approximately 90 min at 37°C. The *oriC*/*ter* ratio was determined by real‐time quantitative PCR. Data are shown as the mean ± SD of three replicates. A significant difference in mRNA was observed between mRNA of the strain expressing QnrB and the strain lacking QnrB (two‐sided Student’s *t*‐test. ^***^ *p* < 0.001). The data shown are representative of three independent experiments. TRIM, trimethoprim; Tet, tetracycline. C. Overexpression of QnrB induces DNA replication stress in *E. coli*. Induction of *E. coli* BW25113 cells harboring pBAD‐*qnrB* or pBAD was performed at an OD_600_ of 0.3 by adding arabinose (0.5% w/v; red column). An untreated culture served as the negative control (blue column). The cells were harvested 40 min after treatment. Replication stress (*oriC/ter* ratio) was measured by real‐time quantitative PCR. Data are shown as the mean ± SD of three replicates. A significant difference in the *oriC*/ter ratio was observed between the strain expressing QnrB and the strain lacking QnrB (two‐sided Student’s *t*‐test. ^****^ *p < *0.0001). The data shown are representative of five independent experiments. [Colour figure can be viewed at wileyonlinelibrary.com]

To confirm that QnrB promotes DNA replication stress, we performed a marker frequency analysis of a native clinical *K. pneumoniae* KP48 strain. The strain was treated with various concentrations of CIP (0, 32, 128, or 192 mg/l) to induce different levels of QnrB expression (Da Re *et al.*, 2009; Wang *et al.*, 2009), and the cells were harvested 90 min later. Genomic DNA and RNA were then extracted, and the *oriC/ter* ratio and *qnrB* mRNA level were determined. Compared with that in non‐treated cells, the *oriC/ter* ratio of the KP48 strain was significantly higher in cells treated with all three concentrations of CIP (3.61 ± 0.14, 12.21 ± 0.17, 11.74 ± 0.14, and 10.63 ± 0.23 at 0, 32, 128, and 192 mg/l respectively) (Fig. 3A left panel). Similarly, the *qnrB* mRNA level was increased significantly by CIP treatment of the KP48 cells (1.28 ± 0.12, 9.40 ± 0.14, 15.56 ± 0.16, and 11.74 ± 0.14 at 0, 32, 128, and 192 mg/l respectively) (Fig. 3A right panel). These results show that the CIP‐induced increase in *qnrB* mRNA expression was accompanied by increased DNA replication stress.

To determine whether increased QnrB expression directly leads to DNA replication stress, we compared the *oriC/ter* ratios in CIP‐treated *K. pneumoniae* pACYC‐*qnrB*/KP49 and pACYC*/*KP49 strains. After treatment with CIP (128 mg/l) for 90 or 120 min, the *oriC/ter* ratios in pACYC‐*qnrB*/KP49 cells were 13.58 ± 0.17 and 12.07 ± 0.07, respectively, whereas those in pACYC/KP49 cells were 10.85 ± 0.27 and 8.67 ± 0.09, respectively (Fig. 3C), indicating that QnrB enhances the DNA replication stress caused by CIP. Next, we measured the *oriC/ter* ratio in *K. pneumoniae* pACYC‐*qnrB*/KP49 and pACYC/KP49 strains treated with two other antibiotics: TRIM and tetracycline. As mentioned earlier, previous studies showed that TRIM blocks dihydrofolate reductase and causes thymineless death by inducing SOS responses and DNA replication stress (Fonville *et al.*, 2010). As expected, TRIM induced *qnrB *mRNA expression (Fig. S3) and increased DNA replication stress. The *oriC/ter* ratios in pACYC‐*qnr*/KP49 and pACYC/KP49 cells were 11.50 ± 0.05 and 6.71 ± 0.09 respectively (Fig. 3B). Tetracycline, a protein synthesis inhibitor that does not increase the *oriC*/ter ratio (Slager *et al.*, 2014), did not significantly induce *qnrB* mRNA expression (Fig. S3). Furthermore, the *oriC/ter* ratio was comparable in tetracycline‐treated pACYC‐*qnrB*/KP49 and pACYC/KP49 cells (4.00 ± 0.23 and 3.56 ± 0.17 respectively) (Fig. 3B). These findings indicate that QnrB can induce DNA replication stress.

Subsequently, we examined the effect of QnrB on DNA replication stress in an *E. coli* BW25113 strain expressing N‐terminally His‐tagged QnrB from the pBAD plasmid (pBAD‐*qnrB*), in which the *qnrB* gene was under the control of the P_araC_ promoter. In this strain, L‐arabinose and glucose induced and inhibited *qnrB* expression respectively. The *oriC/ter *ratio was 5.82 ± 0.04 in cells grown in LB containing L‐arabinose, and 2.58 ± 0.16 in cells grown in glucose‐containing medium (Fig. 3C). In a negative control strain carrying the empty pBAD33 vector (pBAD/BW25113), the *oriC/ter *ratio was 2.60 ± 0.04 in L‐arabinose‐containing medium and 2.32 ± 0.11 in glucose‐containing medium (Fig. 3C). Overall, these findings confirm that QnrB promotes DNA replication stress.

### QnrB‐induced DNA replication stress is independent of DNA gyrase protection

QnrB‐mediated replication stress could be related to the inhibition of DNA gyrase activity and the subsequent effect on DnaA‐dependent replication initiation (Fuller and Kornberg, 1983; Schnos *et al.*, 1988; Samadpour and Merrikh, 2018). However, as described above, TRIM, which exerts its effects independently of gyrase, induced QnrB‐mediated DNA replication stress, resulting in an increased mutation rate (Figs 2 and 3, and Table S1). Therefore, we tested the hypothesis that QnrB can cause DNA replication stress without the involvement of gyrase. DNA replication stress can affect DNA replication and the segregation of chromosomes (Passerini and Storchova, 2016; Zhang *et al.*, 2018), thus influencing the DNA content of cells (Taylor *et al.*, 2005). Consequently, we examined the genomic DNA and plasmid contents of two *E. coli* strains expressing (pQE‐*qnrB*/BW25113) or lacking (pQE/BW25113) QnrB. As predicted, the genomic DNA and plasmid contents of the pQE‐*qnrB*/BW25113 cells were 1.8 ± 0.3 and 2.8 ± 0.8 times higher, respectively, than those of the pQE/BW25113 cells. These results indicate a strong correlation between increased plasmid content and QnrB‐mediated DNA replication stress.

**Figure 4:**
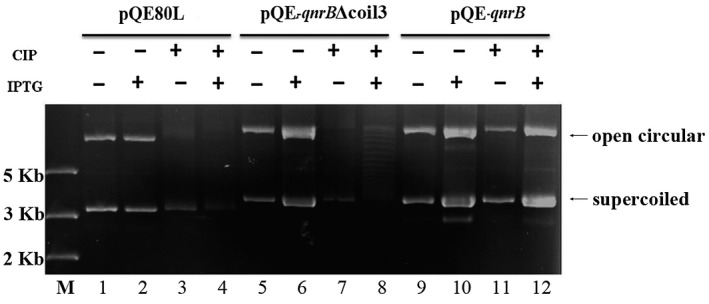
QnrB promotes plasmid propagation in a manner that is independent of DNA gyrase protection. *E. coli* strains harboring pQE80L (empty vector), pQE‐*qnrB* or pQE‐qnrBΔcoil3 were cultured to an OD_600_ of 0.6 in LB at 25°C, and then 0.5 mM IPTG and/or 5 mg/l CIP was added. After culture for 1 h, the plasmids were isolated and dissolved in 50 μl of water. Equal volumes were analyzed by gel electrophoresis. M, DNA marker.

QnrB has eight complete coils, each of which comprises a right‐handed β helix, two extruding loops that are important for protection against CIP, and a C‐terminal tail that is required for dimer formation (Shah and Heddle, 2014). We constructed various QnrB mutants containing deletions of coil 2, coil 3, coil 4, both loops, the C‐terminal amino acid tail or the translation start site (designated Δcoil2, Δcoil3, Δcoil4, Δloop, ΔC‐term or ΔN‐term respectively; the corresponding regions are shown in Fig. S4), and expressed the mutant or wild‐type QnrB proteins in *E. coli*. As seen in cells expressing wild‐type QnrB (Fig. 4, lane 10), IPTG induction increased the plasmid content of cells expressing QnrBΔcoil3 (Fig. 4 lane 6), indicating an increase in DNA replication stress. By contrast, the plasmid contents of *E. coli* cells harboring empty vector (pQE80L) or the other QnrB mutants were not increased after IPTG induction (Fig. 4, lane 2; and Fig. S4).

Quinolones form a complex with DNA and gyrase, and the gyrase‐cleaved DNA cannot be relegated (Hooper and Jacoby, 2015). QnrB is a DNA mimic that is thought to protect DNA gyrase against quinolone damage and reduce quinolone‐mediated DNA cleavage (Merens *et al.*, 2009; Shah and Heddle, 2014). Monitoring plasmid cleavage using agarose gels can be used to detect the protective effects of QnrB on gyrase. Following CIP treatment, plasmid cleavage was observed in *E. coli* cells expressing pQE‐*qnrB *(Fig. 4, lane 12), but not in those expressing the empty pQE80L vector (Fig. 4, lane 4). In addition, plasmid cleavage was not observed in cells expressing pQE‐*qnrB*Δcoil3 (Fig. 4 lane 8). These results indicate that QnrBΔcoil3 increases DNA replication stress but has no protective effect on gyrase (Fig. 4). In addition, glutathione S‐transferase pull‐down experiments showed that intact GST‐QnrB interacted with His_6_‐GyrA/GyrB, but GST‐QnrBΔcoil3 did not (Fig. S4). Overall, these findings indicate that QnrB can increase DNA replication stress regardless of an interaction with gyrase.

### QnrB interacts with DnaA

**Figure 5:**
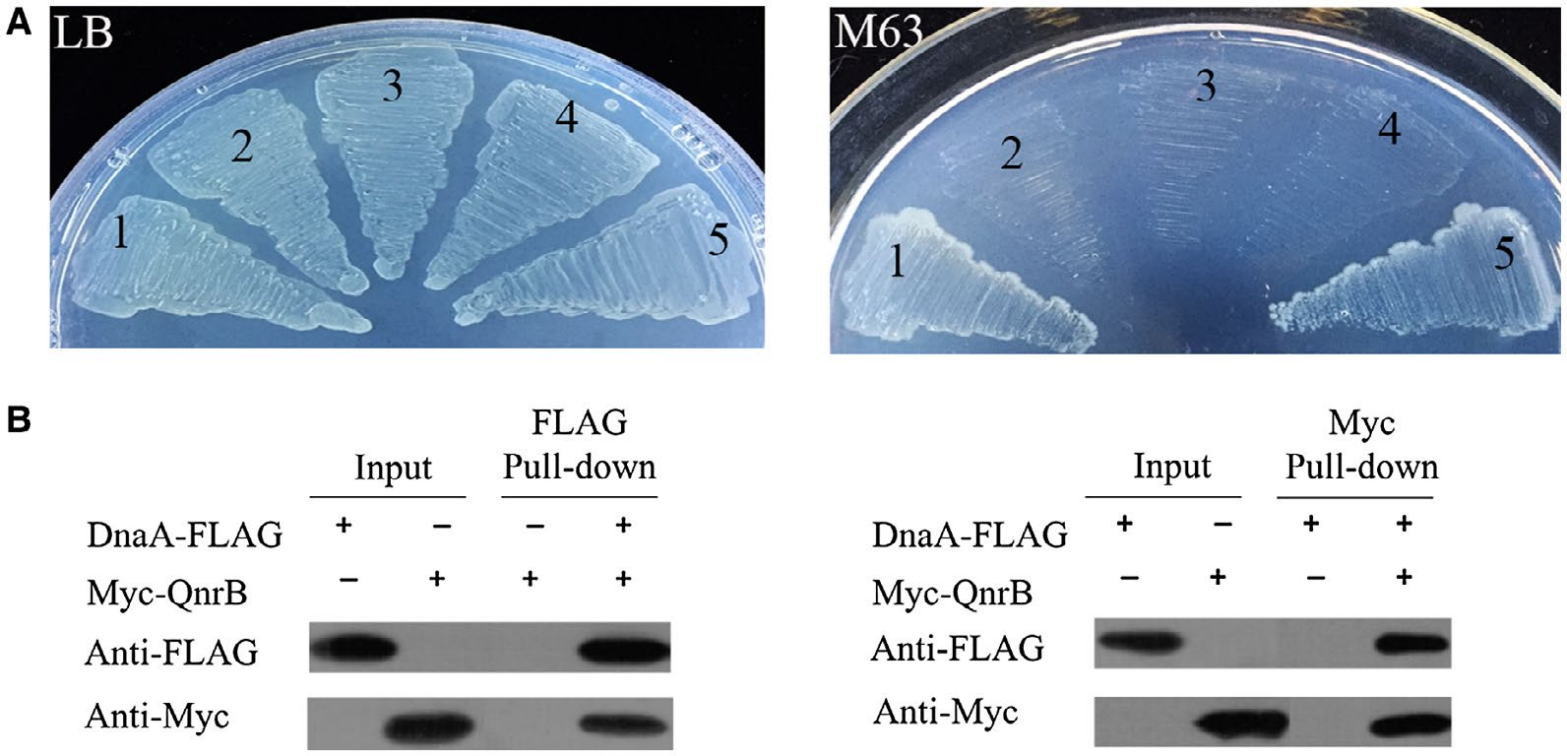
QnrB interacts with DnaA. A. Bacterial two‐hybrid system analysis showing that QnrB associates with DnaA. *E. coli* cells were co‐transformed with the pUT18c‐zip and pKT25‐zip plasmids (1) as a positive control. Cells harboring the pUT18c and pKT25 (2), pUT18c‐*qnrB* and pKT25 (3), and pUT18c and pKT25‐*dnaA* (4) plasmid pairs were included as negative controls. Cells harboring pUT18c‐*qnrB* and pKT25‐*dnaA* were used to detect the interaction between QnrB and DnaA (5). Bacteria were cultured on LB containing 100 mg/l ampicillin (Amp) and 50 mg/l kanamycin (Kan) (left panel), or M63 containing 0.2% maltose (right panel). B. Affinity tagged pull‐down assays to evaluate the interaction between QnrB and DnaA. The proteins were induced and purified from BL21(DE3) cells expressing His_6_‐DnaA‐FLAG or His_6_‐Myc‐QnrB. The purified proteins were diluted 100‐fold, and 2 μl samples were used as the input. The interactions were determined by western blotting using an antibody against FLAG (for DnaA) or Myc (for QnrB). Data represent the results of three independent experiments. [Colour figure can be viewed at wileyonlinelibrary.com]

DNA replication is strictly regulated to ensure that genetic material is accurately inherited by offspring. In all organisms, the DNA replication initiation protein binds the replication origin to initiate DNA replication. In *E. coli*, DnaA forms a complex with *oriC*, resulting in the unwinding of *oriC* to initiate DNA replication (Ozaki and Katayama, 2012; Ozaki *et al.*, 2012; Zorman *et al.*, 2012; Katayama *et al.*, 2017; Hansen and Atlung, 2018). Since QnrB was found to upregulate the expression of *oriC*‐proximal genes (Fig. 2) and increase the *oriC/ter *ratio (Fig. 3), we hypothesized that it might affect the formation of the DnaA‐*oriC* complex. To test this hypothesis, we examined the interaction between DnaA and QnrB using a bacterial two‐hybrid system. The coding regions of *qnrB* and *dnaA* were cloned into pUT18c and pKT25 to generate pUT18c‐*qnrB* and pKT25‐*dnaA*, respectively, which were co‐transformed into the *E. coli* BTH101 strain. We evaluated the interaction of QnrB with DnaA by monitoring the growth status of the co‐transformed cells on M63 medium containing maltose. Similar to the positive control strain (pKT25‐zip/pUT18c‐zip; Fig. 5A(1)), the pKT25‐*dnaA*/pUT18c‐*qnrB* strain (Fig. 5A(5)) had a growth advantage over the negative control strains (pKT25/pUT18c, pUT18c‐*qnrB*/pKT25 and pUT18c/pKT25‐*dnaA*; Fig. 5A(2–4)). The bacterial two‐hybrid assay showed a physical interaction between QnrB and DnaA (Fig. 5A). To confirm this result, we expressed and purified His_6_‐DnaA‐FLAG and His_6_‐Myc‐QnrB in *E. coli* BL21 (DE3) cells. As a control, we also expressed Myc‐ or FLAG‐tagged MSMEG_2415. A pull‐down analysis was then performed between QnrB and DnaA, QnrB and MSMEG_2415, and DnaA and MSMEG_2415 (Figs 5B and S5). QnrB interacted directly with DnaA (Fig. 5B), while neither DnaA nor QnrB interacted with MSMEG_2415 (Fig. S5).

Subsequently, we examined the interaction between QnrB and *oriC* using MST analyses of protein‐protein and protein‐DNA interactions (Wienken *et al.*, 2010; Lippok *et al.*, 2012). The Kd of DnaA for double‐stranded (ds) *oriC* was 18.6 ± 3.8 nM, which is consistent with that reported in previous studies (Kd = 4–20 nM) (Schaper and Messer, 1995; Leonard and Grimwade, 2015; Grimwade *et al.*, 2018). QnrB did not interact with ds‐*oriC* or truncated ss‐*oriC *(F1–F4) (Fig. S6). Taken together, our results indicate that QnrB regulates the formation of the DnaA‐*oriC* complex by interacting with DnaA rather than *oriC*.

### QnrB facilities the formation of the DnaA‐oriC open complex and over‐initiation of DNA replication

Next, we investigated how QnrB affects the formation of the DnaA‐*oriC* complex, leading to DNA replication stress. DnaA is a DNA‐binding protein that binds to the *oriC* region and initiates DNA replication (Kaguni, 2011). QnrB is a DNA mimic protein that regulates DNA‐protein interactions (Wang *et al.*, 2014). Our results described above indicated that QnrB interacts with DnaA but not *oriC *(Fig. S6), inferring that QnrB affects the affinity of DnaA for *oriC*. The Kd of DnaA for ssDNA differs depending on the specific DNA motif (Katayama *et al.*, 2017), so we used MST to measure the affinity of DnaA for various *oriC* fragments in the presence or absence of QnrB (Wienken *et al.*, 2010; Lippok *et al.*, 2012). First, we examined the effect of QnrB on the affinity of DnaA for double‐stranded (ds) *oriC *and *oriC‐*truncates. In the absence of QnrB, the Kd values of DnaA for ds‐*oriC* and the ds‐*oriC* duplex‐unwinding element‐R1 (F1) fragment were 18.6 ± 3.8 nM and 0.13 ± 0.05 μM respectively. In the presence of QnrB, these values were 22.6 ± 1.5 nM and 0.22 ± 0.08 μM, respectively (Fig. S7), indicating that QnrB has little or no effect on the affinity of DnaA for ds‐*oriC/oriC‐*truncates (Fig. S7A and B).

**Figure 6:**
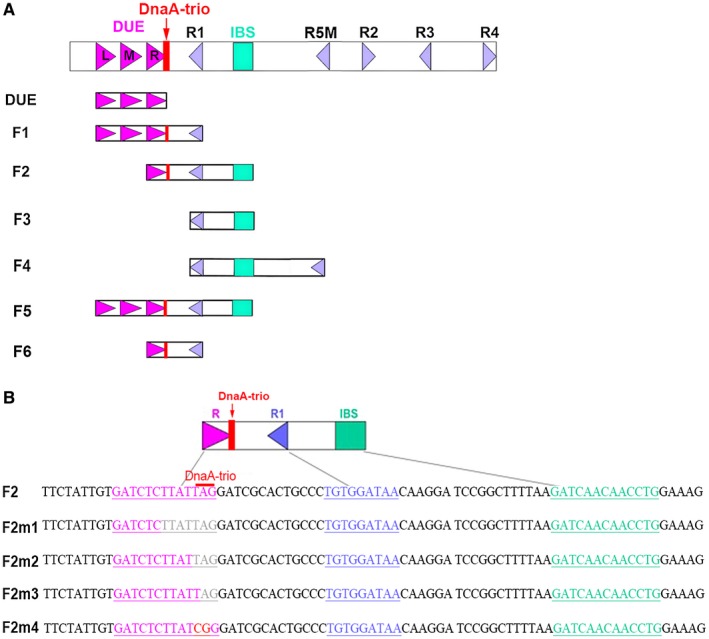
Features of *oriC* mutations. A. Structure of *oriC* showing the AT‐rich 13 bp DUE regions (purple arrows), DnaA boxes (blue triangles), DnaA‐trio (red rectangle) and IBS (green rectangle). DUE, F1, F2, F3 and F4 indicate the truncated *oriC* constructs. B. Overview of the *oriC* fragments containing mutations in the replication origin element. A portion of the DUE‐R 13 bp fragment is indicated in pink. DnaA box R1 is indicated in blue, and the IBS is indicated in green. Mutation sites are indicated in red, and deletion sites are indicated in light gray. The DnaA‐trio is indicated by a red line. [Colour figure can be viewed at wileyonlinelibrary.com]

Previous studies showed that binding of DnaA to ssDNA plays an important role in DNA replication initiation (Duderstadt *et al.*, 2011; Katayama *et al.*, 2017). DnaA binds the *oriC *region containing the DUE and DnaA‐binding sites (DnaA boxes) (Fig. 6A), unwinds the double‐stranded *oriC* DUE, and provides a ssDNA region for DnaB helicase loading, which is required for further DNA replication (Katayama *et al.*, 2017). In addition, other studies showed that the *oriC* DUE‐M, DUE‐R, R1, IHF‐binding site (IBS) and R5M motifs may play important roles in binding of DnaA to ssDNA and in DNA replication initiation (Katayama *et al.*, 2017; Sakiyama *et al.*, 2017). The *Bacillus subtilis oriC* contains a DnaA‐trio motif near DUE‐R, which is essential for binding of DnaA to ssDNA. However, the role of the putative DnaA‐trio in *E. coli *has not yet been identified. Therefore, we synthesized a series of 6‐FAM‐labeled ss‐*oriC* fragments carrying different motifs (termed DUE and F1‐6; Fig. 6A and Table S3), and measured the affinity of DnaA for these fragments in the presence and absence of QnrB. As shown in Tables 1 and 2, QnrB increased the affinity of DnaA for the F2 ss‐*oriC* fragment containing DUE‐R, DnaA‐trio and IBS, but had no effect on the affinity of DnaA for fragments lacking these motifs (Fig. 6B and Tables 1 and 2). The TTATT sequence in DUE‐R is a specific ssDNA‐binding site of DnaA, and the DnaA‐trio sequence is located within the DUE‐R motif (Fig. 6B); therefore, we could not conclude that the DnaA‐trio motif is necessary to promote the formation of the DnaA‐*oriC* complex by QnrB.

**Table 1:** Microscale thermophoresis analyses of the affinity of DnaA for *oriC *with and without QnrB.

	DnaA motif							QnrB effect
	DUE‐L	DUE‐M	DUE‐R	DnaA‐trio	R1	IBS	R5M	
DUE	+	+	+					−
F1	+	+	+	+	+			+
F2			+	+	+	+		+
F3					+	+		−
F4					+	+	+	−
F5	+	+	+	+	+	+		+
F6			+	+	+			−

**Table 2:** Microscale thermophoresis analyses to determine the importance of the DnaA‐trio to the DnaA‐*oriC* complex in the presence of QnrB.

	Sequence	DnaA‐trio	QnrB effect
F2	GATCTCTTATTAG	+	+
F2m1	GATCTC• • • • • • •	−	−
F2m2	GATCTCTTAT• • •	−	−
F2m3	GATCTCTTATT• •	−	+
F2m4	GATCTCTTATCGG	−	+

To elucidate the importance of the DnaA‐trio motif to DnaA‐*oriC* complexes, we synthesized four 6‐FAM‐labeled F2 derivatives containing mutations in the DNA‐trio motif (ss‐F2m1–4) and performed MST analyses to measure the Kd values of DnaA for these derivatives in the presence of QnrB. When the TTATTAG or TAG sequence was deleted (ss‐F2m1 or ss‐F2m2 respectively; Fig. 6B), both the DUE‐R and DnaA‐trio motifs were incomplete, and QnrB had no effect on the affinity of DnaA for these derivatives (Table 2). By contrast, QnrB affected the affinity of DnaA for the ss‐F2m3 derivative (Table 2), in which the AG sequence was deleted, resulting in disruption of the DnaA‐trio and DUE‐R motifs but retention of the TTATT sequence. Similarly, QnrB affected the affinity of DnaA for the ss‐F2m4 derivative (Table 2), in which the TA sequence was replaced by CG (F2m4). Overall, these results showed that the DnaA‐trio motif is not required for QnrB to promote the affinity of DnaA for *oriC* (Tables 1 and 2). Our results also showed that QnrB increases the affinity of DnaA for ss‐*oriC *fragments carrying DUE‐R and IBS motifs.

The AAA + domain of DnaA contributes to ssDNA formation during *oriC* unwinding (Fujikawa *et al.*, 2003; Duderstadt *et al.*, 2011; Richardson *et al.*, 2016); therefore, we examined the effect of QnrB on the ATPase activity of DnaA. Unlike QnrB, DnaA has ATPase activity, but this activity was not affected by QnrB (Fig. S8). Along with the evidence that QnrB does not interact directly with *oriC* (Fig. S6), these results suggest that QnrB promotes the binding of DnaA to ss‐*oriC*, thereby increasing DNA replication initiation (Keyamura *et al.*, 2007).

**Figure 7:**
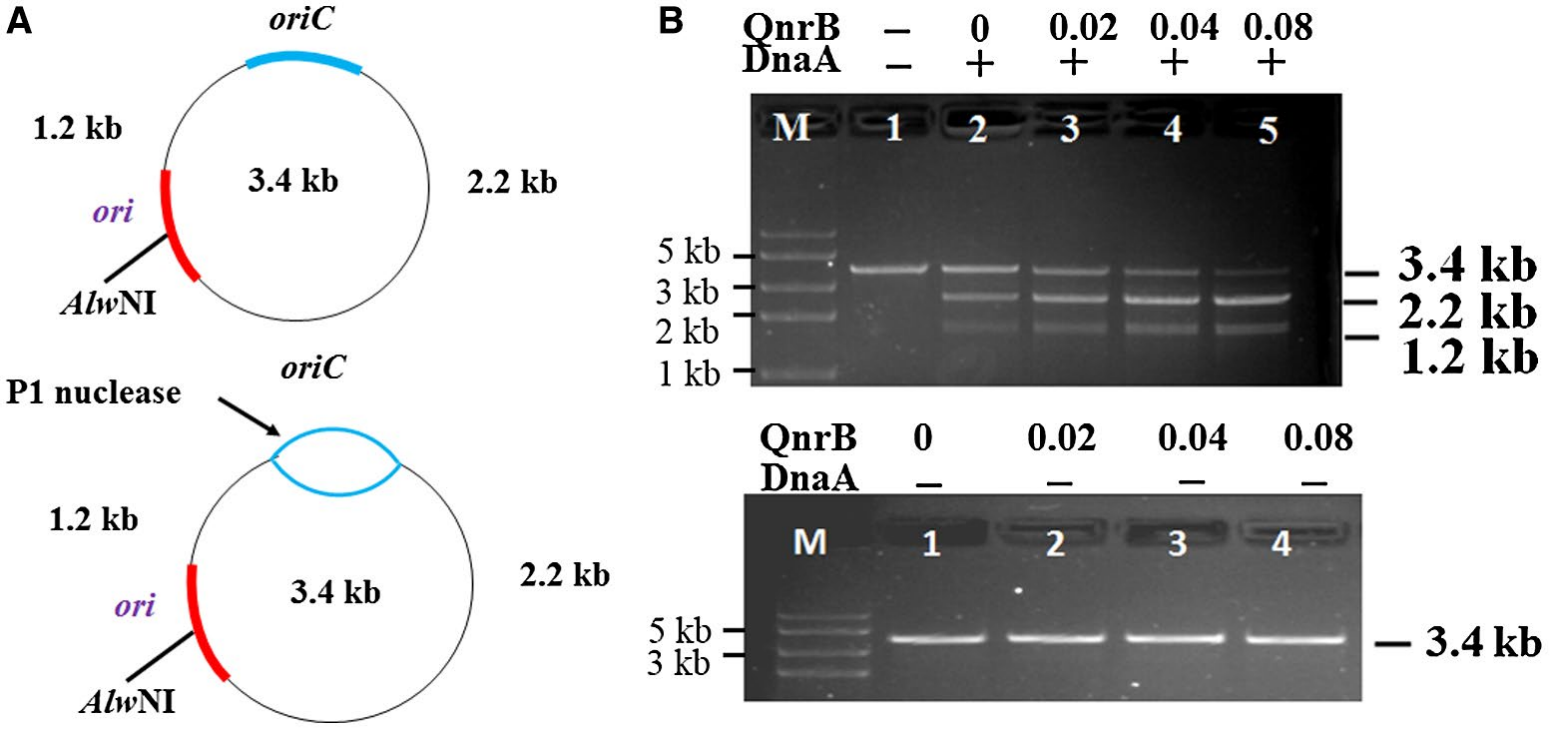
QnrB enhances the formation of the DnaA‐*oriC* open complex. A. Overview of the pUC‐*oriC* plasmid and the *oriC* unwinding assays. DnaA unwinds *oriC* and forms ssDNA. Digestion of the plasmid with P1 nuclease and *Alw*NI produces 1.2 and 2.2 kb fragments. The blue box indicates the *oriC* cloned from *E. coli* BW25113 genomic DNA. The red box indicates the plasmid *ori*. B. QnrB promotes the formation of the DnaA‐*oriC* open complex. Upper panel: The indicated concentrations of QnrB (0‐0.08 nM) were incubated for 5 min at 37°C in buffer containing pUC‐*oriC* (0.5 μg), ATP (1 mM), HU protein (30 ng) and DnaA (0 or 0.04 nmol), and then incubated for 3 min in the presence of P1 nuclease. After digestion with *Alw*NI, the DNA fragments were analyzed by 1% agarose gel electrophoresis. Data are representative of three independent experiments. Lower panel: QnrB had no effect on *oriC* unwinding. The open complex formation was performed in the absence of DnaA. [Color figure can be viewed at wileyonlinelibrary.com]

Next, we performed an *oriC* unwinding assay to investigate whether QnrB promotes the formation of *oriC* open complexes. P1 nuclease specifically degrades ssDNA, and the pUC‐*oriC* plasmid contains a unique *Alw*NI restriction site in the replication origin (*ori*) of the plasmid (Fig. 7A). When pUC‐*oriC* was treated with P1 nuclease followed by *Alw*NI, 2.2 and 1.2 kb fragments were produced (Fig. 7A). In the presence of limited amounts of DnaA, QnrB promoted the formation of the 2.2 and 1.2 kb fragments in a concentration‐dependent manner, indicating increased *oriC* open complex formation (Fig. 7B, upper panel). Similarly, when pUC‐*oriC* was treated with P1 nuclease followed by the *Ssp*I restriction enzyme, the recognition site of which is located in the backbone of pUC‐*oriC*, QnrB increased the formation of the expected 2.3 and 1.1 kb fragments (Fig. S9B). In the absence of DnaA, QnrB did not affect *oriC *unwinding (Fig. 7B, lower panel). Taken together, these results show that QnrB facilitates the formation of an open DnaA‐*oriC* complex, which leads to over‐initiation of DNA replication.

## Discussion

Antibiotic resistance is a global health threat, and plasmid transmission is one way in which bacteria can obtain resistance (San Millan, 2018). Transmitted plasmids cause compensatory mutations that allow bacteria to adapt to their presence; however, the molecular mechanisms underlying this process are not fully understood (Carroll and Wong, 2018). In this study, we found that the plasmid protein QnrB (quinolone resistance protein) increased the bacterial mutation rate by interacting with DnaA, which promoted the DnaA‐*oriC* interaction and formation of the DnaA‐*oriC *interaction, and by supporting the formation of the DnaA‐*oriC* open complex, resulting in over‐initiation of DNA replication. Our findings may explain why QnrB promotes the selection of high‐level quinolone resistance mutants; the increased mutation rate caused by QnrB could increase the chances of selecting bacterial antibiotic resistance mutants (Rando and Verstrepen, 2007). Our studies have delineated a molecular mechanism by which plasmids not only spread antibiotic resistance genes, but also enhance the bacterial mutation rate. An advanced understanding of plasmid‐mediated bacterial antibiotic resistance will provide insights into new strategies for combatting bacterial infections.

Since the discovery of the first plasmid Qnr (Martinez‐Martinez *et al.*, 1998; Tran and Jacoby, 2002), many other plasmid‐borne and chromosomal‐encoded Qnr proteins have been identified in different strains (Tran and Jacoby, 2002; Hooper and Jacoby, 2015). Qnr proteins are distributed widely in bacteria (Table S4) (Jacoby and Hooper, 2013), suggesting widespread existence suggests that they share important biological functions. DnaA is a highly conserved DNA initiator protein that binds to the replication origin and initiates DNA replication in all organisms (Katayama *et al.*, 2017; Hansen and Atlung, 2018). In the current study, we found that QnrB not only interacts with DnaA, but affects its affinity for ss‐*oriC *(Ozaki *et al.*, 2008; Katayama *et al.*, 2017), binding to which requires DUE‐R as well as the IBS element (Tables 1 and 2, and Fig. S7). Given the conserved structure of DnaA and the common specific recognition motifs within the replication origin *oriC*, regulation by Qnr may occur in multiple organisms carrying the *qnr* gene. Furthermore, we found that QnrB results in DNA replication stress (Fig. 3). DNA replication stress is an ‘evolvability factor’ that microbes use to acquire mutations and evolve into drug‐resistant strains. In *Streptococcus pneumoniae*, antibiotics such as CIP over‐initiate DNA replication and trigger bacterial competence, allowing cells to take up antibiotic genes from the environment (Slager *et al.*, 2014). By triggering DNA damage, DNA replication stress plays an important role in cancer development (Di Micco *et al.*, 2006; Bielas *et al.*, 2006; Hills and Diffley, 2014; Wang *et al.*, 2016). These observations suggest that replicative stress‐induced mutagenesis may lead to the development of resistant bacteria and cancer cells. Further examination of the mechanisms involved in this process will aid the development of new strategies for the prevention or treatment of bacterial infections and cancer.

Previous studies showed that *qnrB* expression is controlled by the SOS system and induced by damaging agents (Da Re *et al.*, 2009). In the current study, CIP induced expression in the *qnrB* (Fig. 3) mRNA. In addition, TRIM, but not tetracycline, induced QnrB‐mediated DNA replication stress (Fig. S3). Like CIP, TRIM induced a SOS response (Slager *et al.*, 2014). Furthermore, we found that (i) QnrB leads to DNA replication stress, and (ii) the copy numbers and expression levels of *oriC*‐proximal genes were higher in CIP‐treated cells expressing QnrB than in those lacking QnrB (Fig. 2). In addition, QnrB expression increased the *oriC/ter* ratio in *K. pneumoniae *and *E. coli* strains (Fig. 3). We suggest that these effects are caused by QnrB‐mediated promotion of an open DnaA‐*oriC* complex, resulting in over‐initiation of DNA replication (Fig. 7), which may damage the replication fork and trigger SOS and DNA repair pathways. A recent study also showed that bacteria undergo chromosomal gene mutations to adapt to transmitted plasmids (Loftie‐Eaton *et al.*, 2017). Further studies are required to understand the mechanism by which genes involved in the SOS and DNA repair pathways are involved in QnrB‐mediated bacterial mutation, and how QnrB‐containing plasmids interact with the genome to affect the spread of antibiotic resistance genes.

In conclusion, this study found that the plasmid protein QnrB is a DnaA‐binding protein that promotes DNA replication stress, resulting in an increased bacterial mutation rate. Our results explain how QnrB promotes the accumulation of mutations, including quinolone resistance mutators. In addition, our findings suggest that QnrB produces heterogeneity in bacterial populations by increasing the DNA mutation rate and promoting the ability to survive antibiotic exposures.

## Experimental procedures

### Bacterial strains and growth conditions

All strains and plasmids used in this study are shown in Table S2. Briefly, *E. coli* BW25113 strains harboring pQE‐*qnrB* or QnrB mutants and pBAD‐*qnrB* were used for measurements of genomic DNA/plasmid contents and examination of bacterial growth. The *E. coli* BL21‐Gold (DE3) strain was used for protein expression and purification. All *E. coli* strains were grown in LB supplemented with 50 mg/l kanamycin sulfate or 100 mg/l ampicillin as necessary. The *K. pneumoniae* KP48 strain harboring the pKP048 plasmid was grown in LB containing 32 mg/l CIP from frozen stock, and the *K. pneumoniae* KP49 strain, lacking the pKP048 plasmid was grown in LB.

### Mutation rate examination

A Luria and Delbruck fluctuation analysis (Luria and Delbruck, 1943; Sarkar *et al.*, 1992; Hall *et al.*, 2009) was performed to determine the genomic mutation rates of *K. pneumonia* and *E. coli*. All strains were tested in at least three independent biological replicates. This method is commonly used to measure the spontaneous mutation rate in tested strains, and rifampicin resistance, mediated via mutations in the *rpoB* gene, was used as a marker. *E. coli* BW25113 strains carrying pQE80L (as a blank control), pQE‐*msmeg_2415* (Li *et al.*, 2014) (as a negative control) and pQE‐*qnrB* were used to measure mutation rates. The strains were grown in LB at 37°C overnight, with mixing at 200 rpm. The cultures were reinoculated at 1:100 in LB to an OD_600_ of 0.5, diluted 100‐fold and then reinoculated at 1:100 in 40 ml of LB. After shaking for 6.5 h, a 200‐ml aliquot of bacteria was collected and resuspended in 2 ml of fresh LB. Next, a 50‐µl aliquot of the resuspended culture was plated onto LB plates containing 50 mg/l rifampicin and the number of colonies was counted after incubation at 37°C for 36 h. The initial inoculation number and mutation number were recorded as N0 and M0 respectively.

In *K. pneumonia*, the pACYC/KP49 (Blank), pACYC‐*msmeg_2415*/KP49 (negative control, Ctr) and pACYC‐*qnrB*/KP49 strains were used to measure the mutation rate. The strains were grown in LB at 37°C overnight. The cultures were reinoculated at 1:50 in LB medium to an OD_600_ of 0.5, and then reinoculated at 1:400 in 40 ml of LB medium. Mutation rates were examined following 12 h of growth in the presence of antibiotic. The onefold minimum inhibitory concentration of antibiotic was used for the mutation rate assay. The initial inoculation and mutation colonies were recorded and the final culture number and mutant colonies were counted.

MSS Maximum Likelihood Estimation methods (FALCOR, http://www.keshavsingh.org/protocols/FALCOR.html) (Hall *et al.*, 2009) were used to calculate the mutation rates. The mutation rate was compared in strains with and without QnrB under different treatments. Mutation rates were statistically compared using a two‐tailed *t*‐test.

### Construction of expression vectors

The *dnaA*, *gyrA*, *gyrB*, *hupA* and *hupB* genes were amplified from *E. coli *BW25113 genomic DNA. The *qnrB* gene was amplified from *K. pneumoniae* KP48 strain harboring the pKP048 plasmid, a gift from Dr. Jiang (Jiang *et al.*, 2010), and the PCR fragment was cloned into the pQE80L, pGEX‐5x‐3, pACYCDuet1 and pBAD33 vectors. Deletion mutants, including *qnrB*Δcoil2, *qnrB*Δcoil3, *qnrB*Δcoil4, *qnrB*Δloop, *qnrB*ΔC‐term and *qnrB*ΔN‐term, were constructed using the QuikChange Site‐Directed Mutagenesis Kit (Stratagene, USA). The corresponding deletion positions in *qnrB* are shown in Figure S4. The primers used in this study are shown in Table S3. The constructed vectors were confirmed by DNA sequencing (BGI Company, China).

### Protein expression and purification

QnrB, GyrA, GyrB, DnaA and MSMEG_2415 were overexpressed in *E. coli *BL21‐Gold(DE3) cells. The cells were grown at 37°C, and protein expression was induced by the addition of 0.5 mM IPTG when the cells reached an OD_600_ of 0.5. Subsequently, the cells were collected by centrifugation after 4 h of extended growth at 23°C. Protein purification and concentration measurements were performed as described previously (Zawilak‐Pawlik *et al.*, 2006; Harper and Speicher, 2011; Tao *et al.*, 2013). Details of the protein purification protocol are available on request. The quality of the purified QnrB and DnaA proteins is shown in Figure S10.

### Isolation and curing of the large plasmid from KP48

The *K. pneumoniae *KP48 strain was cultured in LB at 37°C overnight. The cells were then reinoculated at 1:500 into LB containing 5% sodium dodecyl sulfate and grown at 42°C for 10–12 h. Three to five rounds of inoculation and growth were performed. Next, a 50 μl aliquot of culture was diluted 10,000‐fold and plated onto LB plates. Colonies that had lost pKP048 were detected by colony PCR using primers specific for QnrB (see Table S2).

### RNA sequencing

Overnight cultures of KP48 and KP49 cells were diluted 1:100 in LB medium and treated with 256 mg/l CIP for 30 or 90 min when growth reached an OD_600_ of 0.25. The cells were then harvested by centrifugation (11,000 g for 10 min) and frozen. RNA extraction, library preparation and whole‐mRNA sequencing were performed by Novogene (Beijing, China). For RNA sequencing, libraries were generated using the NEBNext Ultra Directional RNA Library Prep Kit for Illumina (NEB, USA). Sequencing of cDNA libraries was performed with an Illumina HiSeq platform, and paired‐end reads were generated. The reference genome and gene model annotation files were downloaded from the genome website. Building of the reference genome index and alignment of clean reads to the reference genome were performed with Bowtie 2–2.2.3 (Langmead and Salzberg, 2012). The results obtained at different times showed a very high correlation (*R* ^2^ > 0.9). The FPKMs (Fragments Per Kilobase of transcripts sequence per Millions base pairs) of genes for the 30 min CIP‐treated group were used when the FPKMs of genes showed the same trend at 30 and 90 min.

### DNA sequencing

For DNA sequencing, overnight cultures of KP49 and KP48 cells were diluted 1:100 in LB containing CIP and grown to an OD_600_ of 0.25 (approximately 60 min). The cells were then harvested by centrifugation (11,000 g for 10 min) and frozen. DNA was fragmented by ultrasonication, and library preparation was performed using the Illumina TruSeq DNA Sample Preparation Kit (Illumina, USA). Pair‐end sequencing was performed on an Illumina HiSeq 2000 system (Illumina, USA). Short reads were assembled using SOAPdenovo (http://soap.genomics.org.cn), a genome assembler specifically developed for next‐generation short‐read sequences (Luo *et al.*, 2012). The algorithm was sensitive to sequencing errors, such that low‐quality reads were filtered, and high‐quality reads were used for *de novo* assembly. The sequences were filtered for low‐quality reads using the DynamicTrim Perl script within SolexaQA. The SOAP GapCloser package was used to close gaps after genome assembly. The genome sequence was uploaded to the Rapid Annotation using Subsystem Technology (RAST) (Aziz *et al.*, 2008) server for annotation.

*E. coli* BW25113 strains harboring pBAD33 or pBAD‐*qnrB* were grown in LB at 37°C overnight. The cultures were then reinoculated at 1:100 in LB supplemented with 1% glucose (w/v) at 30°C. At an OD_600_ of 0.25, the cells were collected by centrifugation at 20°C, and immediately resuspended in LB. L‐arabinose (0.5%) was then added to the resuspended cells, and the cultures were grown with aeration at 30°C for 30 min. Next, 10^8^ bacteria were collected and used for genomic DNA isolation. DNA sequencing was performed as described above.

A number of windows were used to split the reference genome, and these values were used to compute the graphs that plot information across the reference genome. Basically, reads falling in the same window were aggregated in the same bin. The higher the number, the bigger the resolution of the plots but also the longer the data processing time. By default, 400 windows were used. Visualization and statistical analysis were performed using R statistical software (http:www.r-project.org/).

### Real‐time quantitative PCR analyses of the oriC/ter ratio and qnrB mRNA levels

Overnight cultures of *K. pneumoniae *KP48 and KP49 strains were diluted 1:100 in LB containing CIP (0, 32, 128 or 256 mg/l) and grown at 37°C for 90 min. Other samples were treated with CIP (64 mg/l) for 90 or 120 min, or TRIM (0.7 mg/l) or tetracycline (1 mg/l) for approximately 90 min at 37°C. Cells at an OD_600_ of 1.0 (approximately 10^8^ bacteria) were collected and used for extraction of genomic DNA.

*E. coli* BW25113 cells bearing plasmids were grown with aeration in LB supplemented with 1% glucose (w/v) at 37°C. At an OD_600_ of 0.3, the cells were collected by centrifugation at 20°C and immediately resuspended in LB. Glucose (1%) or L‐arabinose (0.5%) was then added to equal portions of the resuspended cells, and the cultures were grown with aeration at 30°C for 40 min.

Genomic DNA was extracted from *K. pneumoniae* and *E. coli* cells using the TIANamp Bacteria DNA Kit. The *oriC/ter *ratio and *qnrB* mRNA levels were determined by quantitative PCR as described previously (Simmons *et al.*, 2004; Felczak and Kaguni, 2009), using the primers listed in Table S3, and data analyses were performed as described previously (Simmons *et al.*, 2004; Felczak and Kaguni, 2009). Each 20 μl sample consisted of 50 ng of cDNA, 2 pmol of each primer (Table S3) and 10 μl of 2 × SYBR Green Supermix. Amplification was performed in an iQ5 Real‐Time PCR Detection System, using the following program: 95°C for 1.5 min; 40 cycles of 95°C for 10 s, 60°C for 15 s and 72°C for 15 s; and then 72°C for 6 min. The real‐time PCR results were calculated as the mean ± SD of three replicates. Overnight cultures of the *K. pneumoniae* *qnrB* ^+^/KP48 strain were used as a reference.

### Examination of plasmid DNA yields in E. coli

Recombinant bacterial strains harboring individual vectors were grown at 37°C overnight in 5 ml of LB containing ampicillin (100 mg/l). Next, 50 µl aliquots of the cultures were cultured to an OD_600_ of 0.6 at 37°C in 5 ml of LB broth containing ampicillin (100 mg/l) and split into two identical sets of cultures. One set of cultures was cultured for 1 h with or without CIP (5 mg/l), and the other set was cultured for 1 h with IPTG (1 mM) and with or without CIP (5 mg/l) at 25°C. All cultures were subjected to plasmid DNA isolation using the Plasmid Miniprep Kit. Equal volumes (15 μl) were directly analyzed by gel electrophoresis or digested with *Bam*HI and then analyzed by the same method.

### Pull‐down and co‐immunoprecipitation assays

For GST pull‐down assays, mixtures of His_6_‐GyrA/GyrB and GST‐QnrB were incubated for 3 h at 25°C, and then incubated with glutathione‐Sepharose beads for 4 h at 4°C. After three washes of the beads with buffer comprising 50 mM Tris‐HCl (pH 8), 150 mM NaCl and 0.1‐0.5% NP‐40, the samples were resuspended in SDS‐PAGE loading buffer and boiled for 5 min before western blotting analysis. Assays were performed with 1 × TBS containing 1 mM PMSF for FLAG‐QnrB and Myc‐DnaA.

### Microscale thermophoresis

MST experiments were performed using a Monolith NT.115 instrument (NanoTemper Inc., Germany), as described previously (Seidel *et al.*, 2012). Fluorescent 6‐FAM‐labeled oligonucleotides were purchased from Sangon Biotech (Shanghai, China). The labeled DNA sequences are shown in Table S3. Once dissolved in water, the oligonucleotides were stored in light‐protected vials at −20°C. A thermal gradient of 4°C was set up over a period of 30 s using an infrared laser with 30% intensity setting. The labeled *oriC* fragments of DNA (200 nM) were incubated with purified ATP‐DnaA at a gradient concentration (ranging from 0 or 0.0012 to 80 μM) in buffer (20 mM HEPES, 50 mM NaCl, 10 mM MgCl_2_, pH 7.6). The MST assay was performed with 30% LED power and 80% MST power. The Kd Fit function of the Nano Temper Analysis Software (v.1.5.41) was used to fit curves and calculate the value of the dissociation constant (Kd). A positive effect was assigned when the ratio of Kd values was larger than 1.5.

### oriC unwinding assays

The *oriC* unwinding assays were performed as described previously (Keyamura *et al.*, 2007), with a few modifications. Briefly, the required amounts of QnrB were incubated for 5 min at 37°C in buffer (20 μl) containing 50 mM Tris‐HCl (pH 7.5), 10 mM magnesium acetate, 8 mM DTT, 100 mg/l BSA, 10% glycerol, 150 mM potassium glutamate, 30 ng of HU protein, 1 mM ATP and the pUC‐*oriC* plasmid (500 ng; 0.22 pmol) in the presence or absence of DnaA (0.04 nmol), and then incubated for a further 3 min in the presence of P1 nuclease (10 U, Sigma). After chloroform extraction and ethanol precipitation, the plasmids were digested with *Alw*NI or *Ssp*I and the DNA fragments were analyzed by electrophoresis on a 1% agarose gel.

### Bacterial growth on LB agar plates

Growth of *E. coli* BW25113 cells harboring the empty pQE80L vector (pQE‐*qnrB*) was evaluated on LB plates supplemented with or without 0.2 mM IPTG at 28°C. Each spot was inoculated with a 2‐μl aliquot from a 10‐fold dilution series of stationary cells (10^−1^‐, 10^−2^‐, 10^−3^‐ and 10^−4^‐fold). Photographs were taken after incubation at 37°C for 24 h.

### Statistical analysis

Statistical analyses were performed using GraphPad Prism 5.0c software. Significant differences were determined by *t*‐tests.

## Author contributions

XL, YZ, XZ, XH, YZ, DL performed all the experiments. KM designed the study and performed the bioinformatics analyses. KM wrote a preliminary draft of the manuscript. KM and AM revised the manuscript. All authors reviewed and approved the manuscript.
