# Supplementary material for: The plasmid‐borne quinolone resistance protein QnrB, a novel DnaA‐binding protein, increases the bacterial mutation rate by triggering DNA replication stress
Source: Mol Microbiol. 2019 Mar 27;111(6):1529–43. doi: 10.1111/mmi.14235 (PMC6617969; doi:10.1111/mmi.14235)
Supplement: Supplementary file 1 [file MMI-111-1529-s001.pdf]

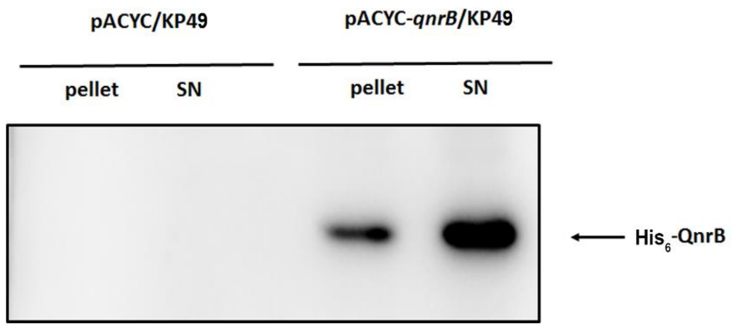

Figure S1 Recombinant QnrB expressed in *K. pneumoniae* pACYC-*qnrB/*KP49. A single clone was inoculated in LB medium at 37ºC and diluted 1:100 in fresh medium until an OD_{600} of 0.8 was reached. Cells were collected and resuspended into 1 ml of PBS. Cells were then transferred into a 2-ml screw cap tube and mixed with 0.5 ml of glass beads. The mixture was vortexed at high-speed using a beads beater. The samples were then centrifuged to separate the pellet and supernatant. The pellets and supernatants were then run on an SDS-PAGE gel and transferred to PVDF membrane. The His-tag antibody was used to probe $_{His66}$-QnrB. *K. pneumoniae* harboring the empty vector pACYC (pACYC/KP49) was used as a negative control. The arrows indicated the position of His_6-QnrB protein. SN, supernatant.

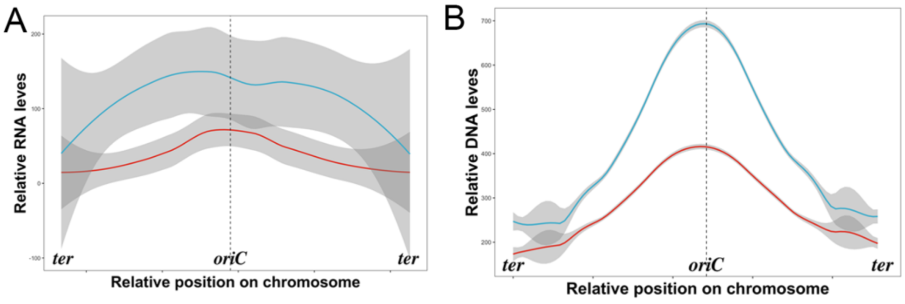

Figure S2 QnrB expression is correlated with upregulation of *oriC*-proximal genes in *E. coli*.

(A) Global transcriptional responses of the pBAD-*qrnB*/BW25113. Strain pBAD-*qnrB*/BW25113 (P_{araC}-*qnrB*) was grown in LB medium with 0.5% arabinose (for inducing QnrB expression) and 1% glucose (negative control) for 30 min. Gene expression (log2) was determined by RNA-seq. FPKM (Fragments Per Kilobase of transcript per Million mapped reads) values for expressed genes (with a log2 fold change > 2 or < -2) are plotted on the chromosome. The error ranges are illustrated by the shaded regions. Red line presents *E. coli* BW25113 without QnrB expression (pBAD/BW25113). Blue line presents *E. coli* with QnrB expression (pBAD-*qnrB*/BW25113).

(B) Genome-wide marker frequency analysis using whole genome sequencing. The medians were read on a sliding window of genes. The shaded regions indicated the coverage derivation from the median of these reads within the window. The red and blue lines indicate the changes in pBAD-*qnrB*/BW25113 growth with glucose and with arabinose, respectively.

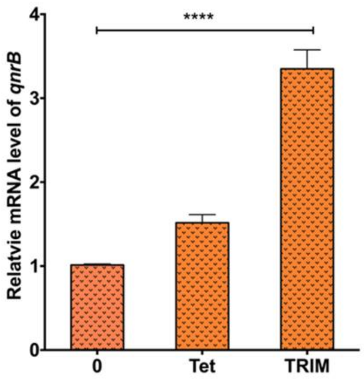

Figure S3 Trimethoprim but not tetracycline induces the *qnrB* mRNA expression. *K. pneumoniae* strain KP48 harboring the QnrB-expressing plasmid pKP048 was treated with trimethoprim (0.7 mg/l) or tetracycline (1 mg/l) for ~90 min at 37 ℃. The *qnrB* mRNA level was measured using real-time quantitative PCR. Data are shown as the mean ± SD of three replicates. **** p <0.0001. The results are representative at least three independent experiments. TRIM, trimethoprim; Tet, tetracycline.

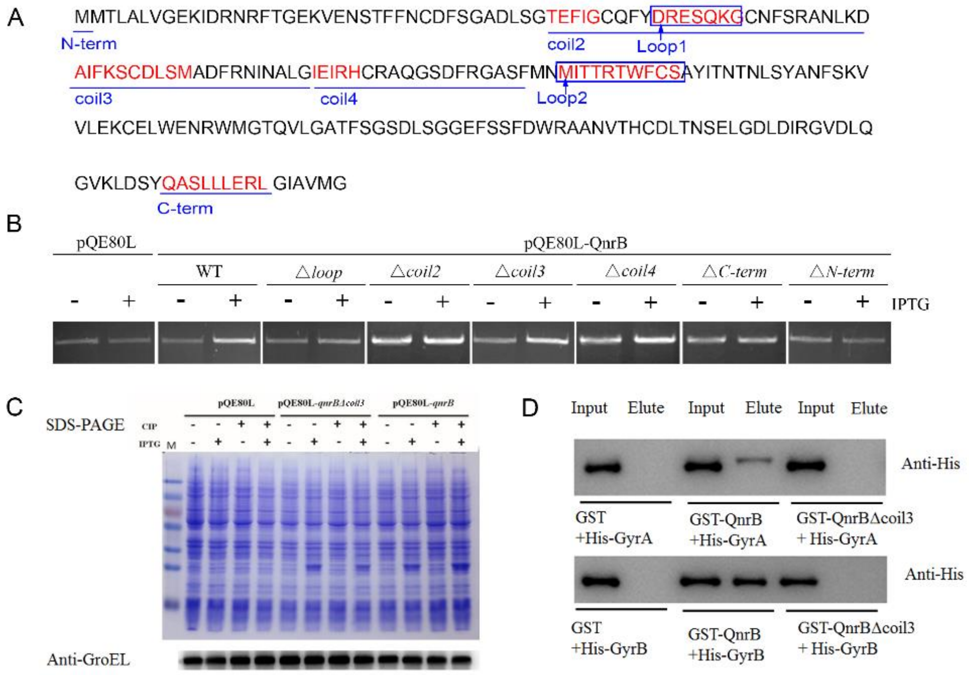

Figure S4 Features of QnrB mutants.

(A) Structure of qnrB. Features of the qnrB sequence are shown, including the N-terminal region, coil2, coil3, coil4, and the C-terminal region. The sequences of loop1 and loop2 are indicated by blue boxes. The deleted sequences are indicated by red text.

(B) Effect of QnrB mutants on DNA replications stress. Plasmid content was used as an indicator as DNA replication stress. Bacterial strains harboring empty vector or plasmids harboring *qnrB* mutants were cultured to OD_{600} of 0.6 in LB at 25°C and 0.5 mM IPTG was added for an additional 1 h of culture. Cells were collected for plasmid isolation and the plasmids were dissolved in 50 µl of water and linearized by treatment with *Eco*RI. Equal volumes (10 µl) were analyzed by gel electrophoresis.

(C) The loading control for measure of QnrB mutants on plasmid contents. Bacterial strains harboring empty vector or plasmids harboring mutated *qnrB* were cultured to OD_{600} of 0.6 in LB broth at 25°C and 0.5 mM IPTG and/or 5 mg/l CIP was added for an additional 1 h of culture. The 10^8 cells were collected and added into 200 µl protein sample buffer and boiled for 10 min and 5 µl samples were analyzed by SDS-PAGE. The loading controls were indicated by using antibody against *E. coli* groEL (hsp60).

(D) GST pull-down analysis of the interaction between GyrA or GyrB and QnrB or its mutant QnrBΔcoil3. His_6-GyrA (up panel) or His_6-GyrB (down panel) and GST-QnrB protein were purified and 0.5 µg purified proteins were incubated for 1 hr. GST beads were used to pull down the complex of GyrA or GyrB and QnrB/ QnrBΔcoil3. Purified proteins (0.1 µg) were used as the input. The interactions were determined by western blotting using an antibody against His_6-tag (for GyrA or GyrB). Data represent the results of three independent experiments.

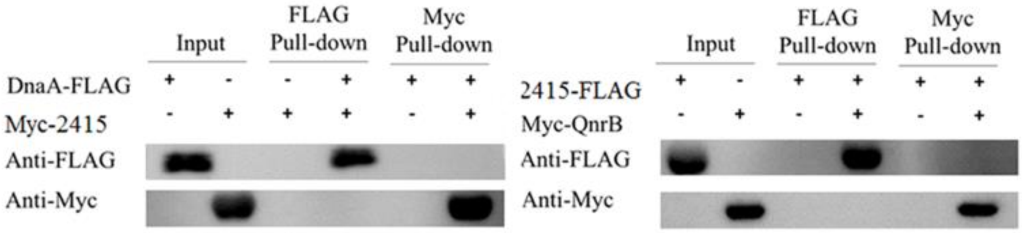

Figure S5 The negative control for the pull-down assay.

Affinity tagged pull-down assays to evaluate the interaction between QnrB/DnaA and MSMEG_2415. The Myc-MSMEG_2415 and MSMEG_2415_FLAG were purified from BL21(DE3) cells. FLAG- and Myc- tagged pull-down were performed. Western bolt was used to determine the interaction. Data represent the results of three independent experiments.

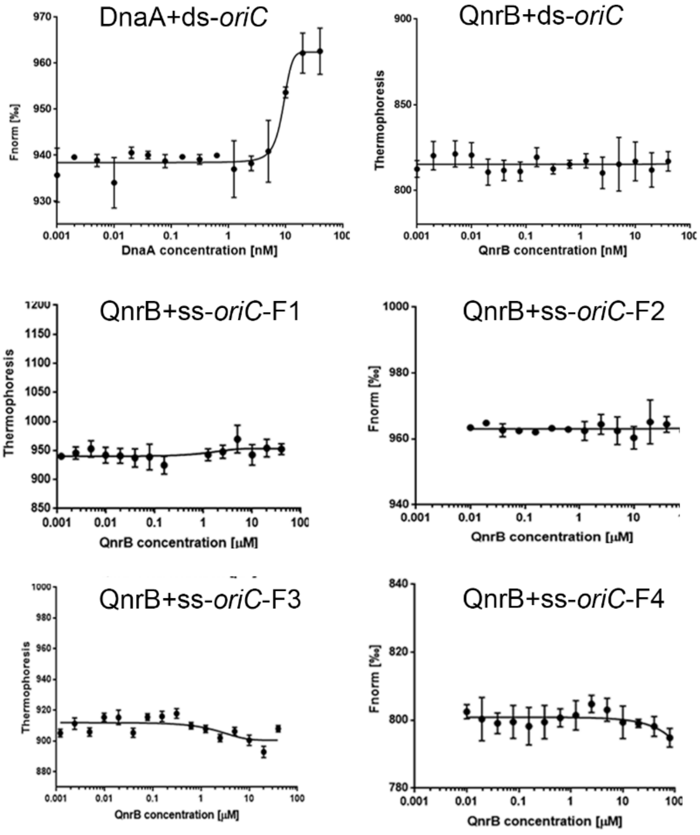

Figure S6 QnrB does not interact with *oriC*.

Microscale thermophoresis (MST) for measurement of the affinity of double-stranded (ds) *oriC* and single-stranded (ss) *oriC* truncates (as shown in Fig. 6) to QnrB. Each measurement was repeated three times. DnaA, DNA replication initiator protein DnaA; ds-*oriC*, double-stranded *oriC*; ss-*oriC*, single-stranded *oriC*.

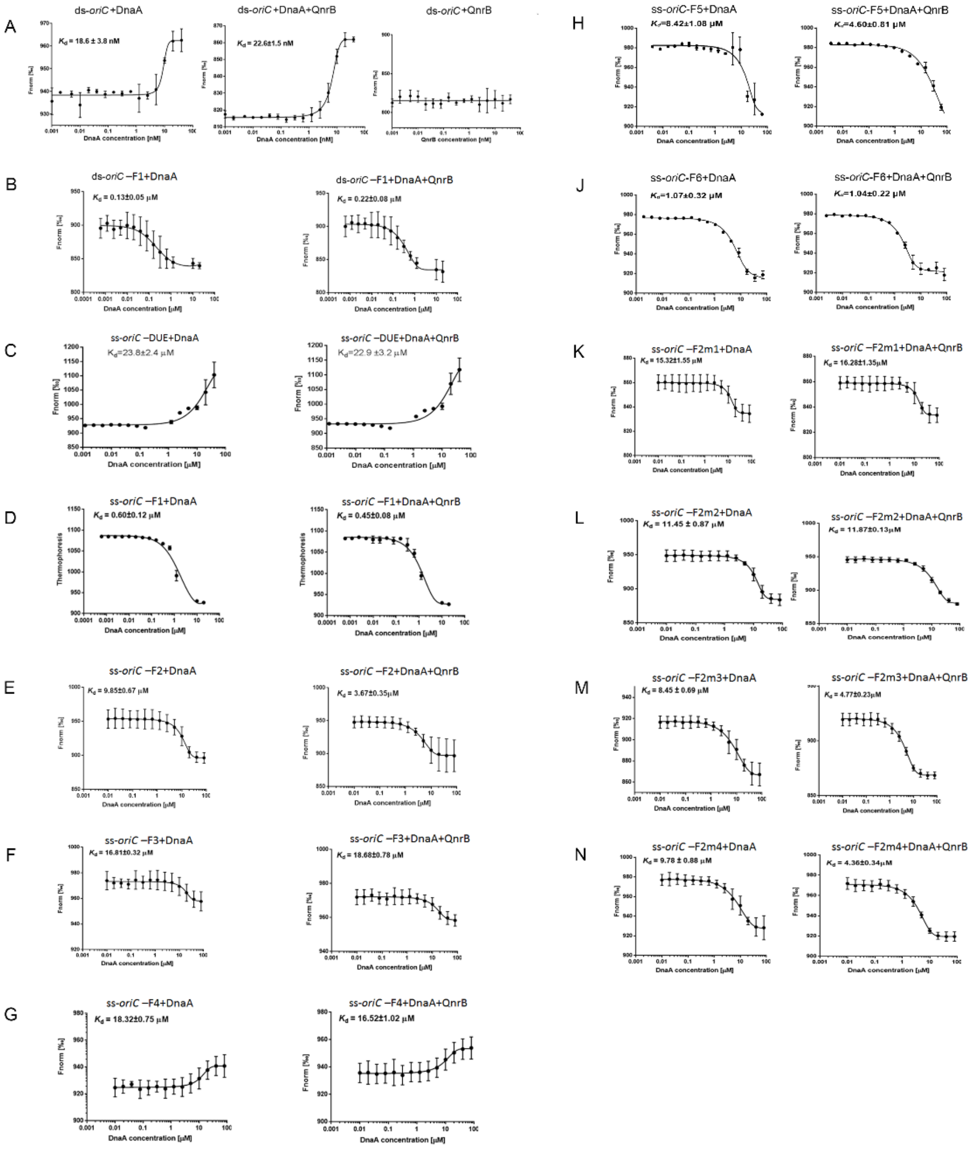

Figure S7 Microscale thermophoresis for measurement of the affinity of DnaA for *oriC*.

To determine the affinity of DnaA for *oriC*, a 16- titration series of one reaction was performed. DnaA from 0, and 0.0012 to 80 μM was incubated with the *oriC*. Each measurement was repeated three times. Nano Temper Analysis software was used for calculation of *Kd* followed the manufacture protocols. A, DnaA and double stranded full-length *oriC* interaction affinity without and with QnrB; B. DnaA and double stranded DUE-R1 region of *oriC* interaction affinity without and with QnrB. C-N DnaA and single stranded *oriC* fragments interaction affinity without and with QnrB.

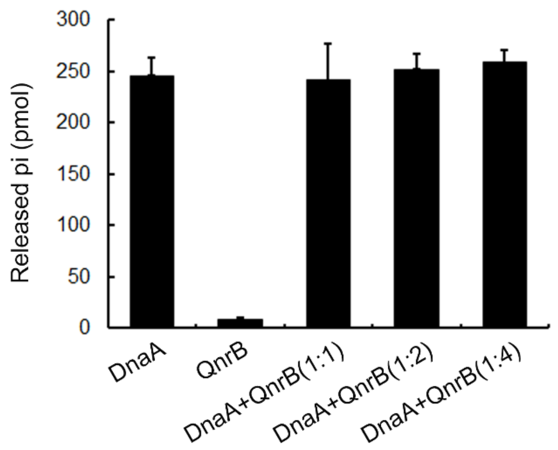

Figure S8 QnrB has no effect on DnaA ATPase activity.

Purified DnaA (10 pmol) and QnrB (10 pmol), and DnaA (10 pmol) mixed with QnrB (10, 20 and 40 pmol) were incubated for 30 min at 37 ℃ and the released free phosphate was determined using a ATPase/GTPase Activity Assay Kit (Sigma, USA) according to the manufacturer's protocol. The assay reaction buffer contains 40 mM Tris·HCl, 80 mM NaCl, 8 mM $Mg(OAc)_2$, 1 mM EDTA, 16.7 nM ATP, pH 7.5 in 240 µl.

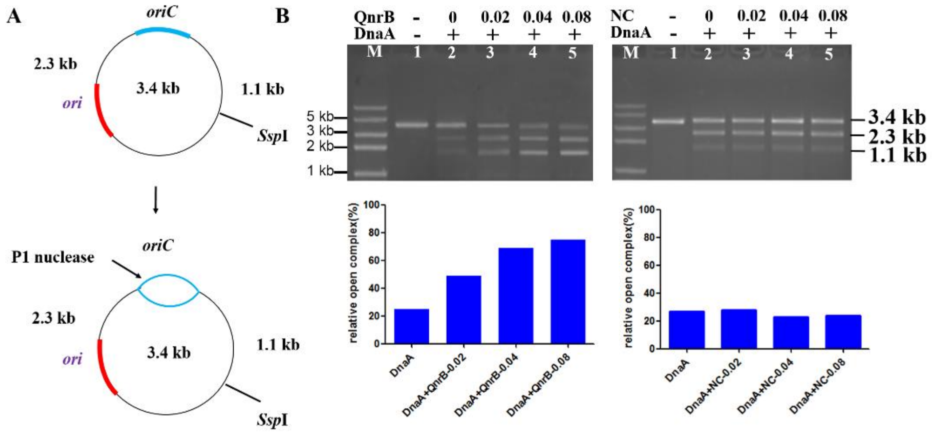

Figure S9 QnrB enhances DnaA–*oriC* open complex formation.

(A) Overview of the pUC-*oriC* plasmid. The blue box indicates *oriC* cloned from *E. coli* BMW25113 genomic DNA. The red box indicates the plasmid *oriC* region. The recognition site of *Ssp*I restriction enzyme is located in the backbone of pUC-*oriC*.

(B) QnrB promotes the formation of an DnaA-*oriC* open complex. The indicated amounts of QnrB (from 0 - 0.08 nM) were incubated for 5 min at 37℃ in buffer containing *oriC* plasmid pUC-*oriC* (0.5 μg), HU protein (30 ng) and ATP (1 mM) in the absence (-) of DnaA, and then incubated for 3 min in the presence of P1 nuclease. After digestion with *Ssp*I, the DNA fragments were analyzed by 1% agarose gel electrophoresis. The intensities of the 2.3-kb and 1.1-kb fragments were quantified using ImageJ software. The relative amounts of the open complex were calculated. Data shown are representative of five independent experiments. BSA was used as a negative control protein to perform the open complex formation assay. Data shown are representative of three independent experiments. NC, negative control.

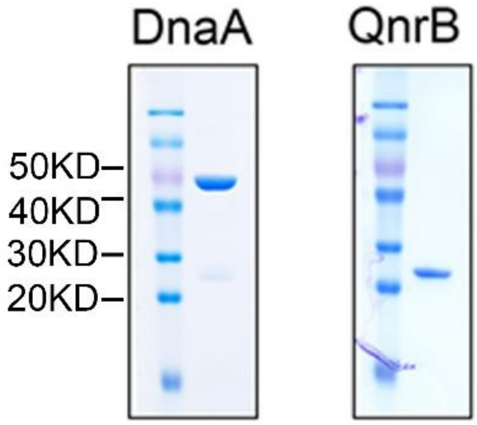

Figure S10. Coomassie blue-stained SDS-PAGE gel of purified DnaA and QnrB recombinant proteins.

Purified proteins were analyzed by SDS-PAGE and visualized by staining with Coomassie brilliant blue.

Table S1. Increased mutation rate was shown in *E. coli* and *K. pneumoniae* strains expressing QnrB

		**MSS-MLE**					**Fold difference**			
		**Mutation Rate (per 10^7)**	**Upper Bound**	**Lower Bound**	**Upper Difference**	**Lower Difference**	**Blank vs Ctr**	**QnrB vs Blank**	**QnrB vs Ctr**	**p value**
	**Blank**	0.03	0.04	0.02	0.01	0.01	1.00			
E. coli	**Ctr**	0.03	0.04	0.02	0.01	0.01				**p=0.0011
	**QnrB**	0.06	0.08	0.04	0.02	0.02		2.01	2.01	***p=0.0007
K. pneumoniae No treatment	**Blank**	0.03	0.04	0.02	0.01	0.01	0.71			
	**Ctr**	0.04	0.05	0.02	0.02	0.01				**P=0.0078
	**QnrB**	0.07	0.10	0.05	0.03	0.02		2.85	2.02	*p=0.0172
K. pneumoniae CIP	**Blank**	0.06	0.09	0.04	0.03	0.02	1.07			
	**Ctr**	0.06	0.08	0.04	0.02	0.02				**P=0.0026
	**QnrB**	0.16	0.20	0.12	0.04	0.04		2.55	2.72	**p=0.0036
K. pneumoniae TRIM	**Blank**	0.34	0.39	0.28	0.06	0.05	1.04			
	**Ctr**	0.32	0.38	0.27	0.05	0.05				***P=0.0008
	**QnrB**	0.80	0.90	0.70	0.10	0.10		2.38	2.47	**P=0.0029

Blank: indicate *E. coli* harboring empty vector pQE80L or *K. pneumoniae* harboring empty vector pACYC
Ctr: indicate strains harboring a vector expressing *qnrB*-irrelated gene, pQE-*msmeg_2415* in *E. coli* and pACYC-*msmeg_2415* in *K. pneumoniae*
QnrB: indicate strain harboring a vector expressing *qnrB*, pQE-*qnrB* in *E. coli* and pACYC-*qnrB* in *K. pneumonia*

Table S2. Bacterial strains and plasmids used in this study

Strains	Description	Source/ comment
***Escherichia coli* strains**		
Escherichia coli DH5α	*E. coli* strain used for cloning	
Escherichia coli BL21-Gold(DE3)	*E. coli* host for protein overexpression	
Escherichia coli BW25113*	*E. coli* host for measure the mutation rate, *oriC/ter* ratio, and growth	
Escherichia coli pQE80L/BW25113	*E. coli* BW25113 harboring pQE80L	This study
Escherichia coli pQE-*qnrB/*BW25113	*E. coli* BW25113 harboring pQE-*qnrB*	This study
Escherichia coli pQE-*qnrB*Δcoil3/ BW25113	*E. coli* BW25113 harboring pQE-*qnrB*Δcoil3	This study
Escherichia coli pQE-*msmeg_2415/* BW25113	*E. coli* BW25113 harboring pQE-*msmeg_2415*	This study
Escherichia coli pBAD-*qnrB/* BW25113	*E. coli* BW25113 harboring pBAD-*qnrB*	This study
Escherichia coli pET-*gyrA/*BL21	*E. coli* BL21-Gold(DE3) harboring pET-*gyrA*	This study
Escherichia coli pET-*gyrB*/BL21	*E. coli* BL21-Gold(DE3) harboring pET-*gyrB*	This study
Escherichia coli pGEX-*qnrB/*BL21	*E. coli* BL21-Gold(DE3) harboring pGEX-*qnrB*	This study
Escherichia coli pGEX-*qnrB*Δcoil3/BL21	*E. coli* BL21-Gold(DE3) harboring pGEX-*qnrB*Δcoil3	This study
Escherichia coli pQE-*dnaA/*BL21	*E. coli* BL21-Gold(DE3) harboring pQE-*dnaA*	This study
Escherichia coli BTH101	*E. coli* host for bacterial two-hybrid assay	This study
Escherichia coli pUT18c-	*E. coli* BTH101 harboring pUT18c-*zip* and pKT25-*zip*	This study

zip+pKT25-*zip/*BTH101		
Escherichia coli pUT18c-*zip*+pKT25-*zip/*BTH101	*E. coli BTH101 harboring pUT18c-zip and pKT25-zip*	This study
Escherichia coli pUT-*qnrB+pKT25* /BTH101	*E. coli* BTH101 harboring pUT-*qnrB* and pKT25	This study
Escherichia coli pUT18c+pKT-*dnaA*/ BTH101	*E. coli* BTH101 harboring pUT18c and pKT-*dnaA*	This study
Escherichia coli pUT-*qnrB*+pKT-*dnaA*/BTH101	*E. coli* BTH101 harboring pUT-*qnrB* and pKT-*dnaA*	This study
***Klebsiella pneumoniae* strains**		
Klebsiella pneumoniae KP48**	a native clinical *K. pneumoniae* from a patient carrying the multidrug-resistance plasmid pKP048	A gift from Dr. Jiang
Klebsiella pneumoniae KP49	a cured strain, whichthe pKP048 plasmid was cured from KP48 using sodium dodecyl sulfate at 42°C.	This study
Klebsiella pneumoniae pACYC/KP49	*K. pneumoniae* harboring the empty vector pACYC	This study
Klebsiella pneumoniae pACYC-*qnrB/*KP49	*K. pneumoniae* harboring the vector pACYC-*qnrB*	This study
Klebsiella pneumoniae pACYC-*msmeg_2415/*KP49***	*K. pneumoniae* harboring the vector pACYC-*msmeg_2415*	This study
**Plasmids**		
pQE80L	Bacterial lacIq vector for expression N-terminally His_6-tagged proteins.	Qiagen
pQE-*qnrB*	Expression His_6-QnrB	This study

pQE-*qnrB*Δcoil3	Expression His_6-QnrBΔcoil3	This study
pQE-*msmeg_2415*	Expression His_6-MSMEG_2415	store in Mi lab
pQE-*dnaA-flag*	Expression His_6-DnaA-FLAG. To examine the interaction with QnrB	This study
pQE-*myc-qnrB*	Expression His_6-Myc-QnrB. To examine the interaction with DnaA	This study
pQE-*dnaA*	Expression of His_6-DnaA. To examine ATPase activity and *oriC* unwinding assay	This study
pBAD33	Cloning expression vector for high-level expression of ORF constructs from the arabinose PBAD promoter	Thermo Fisher Scientific
pBAD-*qnrB*	Expression of QnrB from the PBAD promoter for examination of DNA replication stress	This study
pET23b	Expression vector carrying an N-terminal T7•Tag sequence plus an optional C-terminal His•Tag	EMD Milipore
pET-*gyrA*	Expression vector for expression His_6-GyrA	This study
pET-*gyrB*	Expression vector for expression His_6-GyrB	This study
pACYC-*qnrB*	Expression vector for expression His_6-QnrB in *K. pneumoniae*	This study
pACYCDuet1	Expression vector for expression His_6-fusion protein	Novagen
pGEX-5X-3	Expression vector for expression fusion protein with a GST tag	GE Healthcare
pGEX-*qnrB*	Expression vector for expression GST-QnrB	This study
pGEX-*qnrB*Δcoil3	Expression vector for expression GST-QnrBΔcoil3	This study
pKT25	A multicloning site sequence (MCS) is inserted at the 3' end of T25 to allow construction of in-frame fusions at the C-terminal end of the T25 polypeptide.	Euromedex
pUT18C	This plasmid is designed to create chimeric proteins in which a heterologous polypeptide is fused to the C-terminal end of T18.	Euromedex
pUT-*qnrB*	Expression of QnrB for two-hybrid assay	This study
pKT25-*dnaA*	Expression of DnaA for two-hybrid assay	This study
pUC19	a small, high-copy number E. coli plasmid cloning vector containing portions of pBR322 and M13mp19	NEB
pUC-*oriC*	a vector for *oriC* unwinding assays	This study

* Baba, T et al. (2006) Construction of Escherichia coli K-12 in-frame, single-gene knockout mutants: the Keio collection. Mol. Syst. Biol. 2 2006.0008

** Jiang Y, Yu D, Wei Z, Shen P, Zhou Z, Yu Y. 2010. Complete nucleotide sequence of Klebsiella pneumoniae multidrug resistance plasmid pKP048, carrying blaKPC-2, blaDHA-1, qnrB4, and armA. Antimicrobial agents and chemotherapy 54:3967-3969.
***Li X, Tao J, Hu X, Chan J, Xiao J, Mi K. 2014. A bacterial hemerythrin-like protein MsmHr inhibits the SigF-dependent hydrogen peroxide response in mycobacteria. Frontiers in microbiology 5:800.

Table S3. Oligonucleotide primers used in this study

Primer	Sequence (5'-3')	Purpose
QnrBQE-F	GGAATTCCATATGACGCCATTACTGTATAAAAAAACAGGTA	His$_6$-QnrB constructs
QnrBQE-R	CGCGGATCCCTAACCAATCACCGCGATGC	
QnrB1Δcoil3-F	TTAGTCGTGCGATGCTGAAAATGGCGGATTTTCGCAATTC	His$_6$-QnrBΔcoil3 constructs
QnrB1Δcoil3-R	GAATTGCGAAAATCCGCCATTTTCAGCATCGCACGACTAA	
DnaAQE-F	ACGTGGATCCGTGTCACTTTCGCTTTGGCA	His$_6$-DnaA constructs
DnaAQE-R	GCATGGATCCGGTTGGGATAACGTCCCG	
QE-Myc-QnrB-F	CGCGGATCCGAGCAGAAACTCATCTCTGAAGAGGATCTGATGACGCCATTA CT GTATAA	Myc-QnrB constructs
QE-Myc-QnrB-R	CCCAAGCTTCTAACCAATCACCGCGATGC	
QE-DnaA-F	ACGTGGATCCGTGTCACTTTCGCTTTGGCA	DnaA-FLAG constructs
QE-DnaA-Flag-R	CGGGGTACCTTACTTATCGTCGTCATCCTTGTAATCCGATGACAATGTTCTGATTAA	
QE-2415-Flag-F	CAGGATCCGTGGCCGATTCAAAGCCC	2415-FLAG constructs
QE-2415-Flag-R	GGAAGCTTCTACTTATCGTCGTCATCCTTGTAATCCTTCAGGGCCGAGCC	
QE-Myc-2415-F	CAGGATCCGAGCAGAAACTCATCTCTGAAGAGGATCTGGTGGCCGATTCAAAGCCC	Myc-2415 constructs
QE-Myc-2415-R	GGAAGCTTCTACTTCAGGGCCGAGCCG	
UT18C-QnrB-F	CGCGGATCC CATGACGCCATTACTGTATAA	pUT18C-*qnrB* constructs for bacteria two hybrid
UT18C-QnrB-R	CGGGGTACCCTAACCAATCACCGCGATGC	
KT25-DnaA-F	CGCGGATCC CGTGTCACTTTCGCTTTGGCA	pKT25-*dnaA* constructs for bacteria two hybird

KT25-DnaA-R	CGGGGTACCTTACGATGACAATGTTCTGATTAA	
ACYCduet1- QnrB -F	CGCGGATCCGATGATGACTCTGGCGTTAGT	pACYC-*qnrB* constructs
ACYCduet1-QnrB-R	CCCAAGCTTTTAACCCATGACAGCGATAC	
pBAD33-QnrB-F	CGGGGTACC GGAGTG AAACG ATGACGCCATTACTGTATAA	pBAD-*qnrB* constructs
pBAD33- QnrB-R	CCCAAGCTTCTAACCAATCACCGCGATGC	
pACYCDuet-1(MCSI)	TGAAGTCAGCCCCATACGAT	His$_6$-QnrB sequencing
pBAD-F	ATGCCATAGCATTTTTATCC	pBAD-*qnrB* sequencing
pBAD-R	GATTTAATCTGTATCAGG	
PGEX-5X-QnrB-F	CCGGAATTCATGACGCCATTACTGTATAAAAA	pGEX-*qnrB* constructs
PGEX-5X-QnrB-R	CGCGGATCCCTAACCAATCACCGCGATGC	
pKT25/pUT18-QnrB-F	CGCGGATCC CATGACGCCATTACTGTATAA	pUT-*qnrB* constructs
pKT25/pUT18-QnrB-R	CGGGGTACCCTAACCAATCACCGCGATGC	
pQE80L-QnrB-F	CGCGGATCCATGATGACTCTGGCGTTAGT	pQE-*qnrB* constructs
pQE80L-QnrB-R	CCCAAGCTTTTAACCCATGACAGCGATAC	
pQE80L-QnrBΔloop1-F	GAATTTATCGGCTGTCAGTTCAAAGGGTGCAATTTTAGTCGT	pQE-*qnrB* mutant constructs
pQE80L-QnrBΔloop1-R	ACGACTAAAATTGCACCCTTTGAACTGACAGCCGATAAATTC	
pQE80L-QnrBΔloop2-F	GATTTCCGCGGCGCAAGCTTTTTTTGTAGCGCATATATCACG	pQE-*qnrB* mutant constructs
pQE80L-QnrBΔloop2-R	CGTGATATATGCGCTACAAAAAAAGCTTGCGCCGCGGAAATC	
pQE80L-QnrBΔcoli2-F	CAGGTGCCGACCTGAGCGGCTGTCAGTTCTATGATCGTGA	pQE-*qnrB* mutant constructs
pQE80L-QnrBΔcoli2-R	TCACGATCATAGAACTGACAGCCGCTCAGGTCGGCACCTG	
pQE80L-QnrBΔcoli3-F	TTAGTCGTGCGATGCTGAAAATGGCGGATTTTCGCAATTC	pQE-*qnrB* mutant constructs

pQE80L-QnrBΔcoli3-R	GAATTGCGAAAATCCGCCATTTTCAGCATCGCACGACTAA	
pQE80L-QnrBΔcoli4-F	CGCTGGGCGCAAGCTTTATG	pQE-*qnrB* mutant constructs
pQE80L-QnrBΔcoli4-R	CATAAAGCTTGCGCCCAGCG	
pQE80L-QnrBΔN-term-F	CATCACCATCACGGATCCACTCTGGCGTTAGTTGG	pQE-*qnrB* mutant constructs
pQE80L-QnrBΔN-term-R	CCAACTAACGCCAGAGTGGATCCGTGATGGTGATG	
pQE80L-QnrBΔC-term-F	GCGTCAAACTGGACAGCTACATCGCTGTCATGGGTTAAAAGCTT	pQE-*qnrB* mutant constructs
pQE80L-QnrBΔC-term-R	AAGCTTTTAACCCATGACAGCGATGTAGCTGTCCAGTTTGACGC	
pKT25-b3702-F	CGCGGATCC CGTGTCACTTTCGCTTTGGCA	pKT25-*dnaA* constructs
pKT25-b3702D4-R	CGGGGTACCTTACGATGACAATGTTCTGATTAA	
pUT18CF	TGCCGGCGTCACCCGGATTG	pUT-*qnrB* sequencing
pUT18CR	ACTATGCGGCATCAGAGCAG	
pKT25F	GACCGATTACCTGGCGCGCAC	pKT25-*dnaA* sequencing
pKT25R	GTTTTCCCAGTCACGACGTTG	
EgyrBexF	GCAGCATATGTCGAATTCTTATGACTC	*E. coli* GyrB expression
EgyrBexR	ACGCGGATCCTTACAGATCTTCTTCAGAAATAAGTTTTTGTTCAATATCGATATTCGCCGCTTTC	
EgyrAexF	CTGCGGATCCAATAATTTTGTTTAACTTTAAGAAGGAGATATACATATGAGCGACCTTGCGAGAG	*E. coli* GyrA expression
EgyrAexR	CAATGCGGCCGCTTCTTCTTCTGGCTCGTCGTC	
K.pn-oriC-qF	ATCGGTGATCCTGGTCCGTA	For q-PCR
K.pn-oriC-qR	TCGTCCGATCTTCTGTGGAT	
K.pn-ter-qF	CAGCCCTTCTGGATAAGCCC	For q-PCR
K.pn-ter-qR	TGATCGGTGCTGTGATGACG	
QnrB-qF	TTCGCGGCGCAAGTTTTATG	For q-PCR
QnrB-qR	GCTCGCACTTTTCCAGTACG	
E.coli-relE-qF	AGACCGGAGCTTAATCTTGTAACAA	For q-PCR

E.coli-relE-qR	ACAGTTGAAAAAGAAGCTGG TTGA	
E.coli-oriC-qF	GCCCTGTGGATAACAAGGAT	For q-PCR
E.coli-oriC-qR	CCTCATTCTGATCCCAGCTT	For q-PCR
Oric-ss-right half	TTATACACAACTCAAAAACTG AACAACAGTTGTTCTTTGGAT AACTACCGGTTGATCCAAGCT TCCTGACAGAGTTATCCACA	For MST analysis
Oric-ss-DUE	TGGGTATTAAAAAGAAGATCT ATTTATTTAGAGATCTGTTCT ATTGTGATCTCTTATTAGGAT	For MST analysis
oric-ss-fragment-1	TGGGTATTAAAAAGAAGATCT ATTTATTTAGAGATCTGTTCT ATTGTGATCTCTTATTAGGAT	For MST analysis
oric-ss-fragment-2	TTCTATTGTGATCTCTTATTAG GATCGCACTGCCCTGTGGATA ACAAGGATCCGGCTTTTAAGA TCAACAACCTGGAAAG	For MST analysis
oric-ss-fragment-3	ATCTCTTATTAGGATCGCACT GCCCTGTGGATAACAAGGATC CGGCTTTTAAGATCAACAACC TGGAAAGGATCATTAAC	For MST analysis
oric-ss-fragment-4	AGGATCGCACTGCCCTGTGGA TAACAAGGATCCGGCTTTTAA GATCAACAACCTGGAAAGGA TCATTAACTGTGAATGAT	For MST analysis
oric-ss-fragment-5	GATCTATTTATTTAGAGATCT GTTCTATTGTGATCTCTTATTA GGATCGCACTGCCCTGTGGAT AACAAGGATCCGGCTTTTAAG ATCAACAACCTGGAAAG	For MST analysis
oric-ss-fragment-6	TTCTATTGTG ATCTCTTATT AGGATCGCACTGCCCTGTGGA TAACAAGGATCCGGCTTTTAA	For MST analysis
oric-ss-fragment-2-m1	TTCTATTGTGATCTCGATCGC ACTGCCCTGTGGATAACAAGG ATCCGGCTTTTAAGATCAACA ACCTGGAAAG	For MST analysis
oric-ss-fragment-2-m2	TTCTATTGTGATCTCTTATCGG GATCGCACTGCCCTGTGGATA ACAAGGATCCGGCTTTTAAGA TCAACAACCTGGAAAG	For MST analysis

oric-ss-fragment-2-m3	TTCTATTGTGATCTCTTATTGA TCGCACTGCCCTGTGGATAAC AAGGATCCGGCTTTTAAGATC AACAACCTGGAAAG	For MST analysis
oric-ss-fragment-2-m4	TTCTATTGTGATCTCTTATTCC GATCGCACTGCCCTGTGGATA ACAAGGATCCGGCTTTTAAGA TCAACAACCTGGAAAG	For MST analysis
ds-oric-MST-for<br>ds-oric-MST-rev	GAGAAAGACCTGGGATCCTG<br>GATCCGGCAGAAGAATGGCT	For MST analysis
ds-oric-due+R1-MST-for<br>ds-oric-due+R1-MST-rev	GGTATTAAAAAGAAGATCTA<br>CTTGTTATCCACAGGGCAGT	For MST analysis
UC19-oriC-F<br>UC19-oriC-R	CCGGAATTCGAGAAA GACCTGGGAT CCTGG<br>AAACTGCAGGATCCGGCAGA AGAATGGCTGG	pUC-*oriC* constructs

Figure S4. Plasmid- and chromosomal- *qnr* genes

	Plasmid-located	Chromosomally-located
qnrA	*Acinetobacter baumannii, C. freundii, E. aerogenes, Enterobacter cloacae, Enterobacter sakazakii, E. coli, Haemophilus parasuis, K. oxytoca, K. pneumoniae, P. mirabilis, P. aeruginosa, Pseudomonas oryzihabitans, Pseudomonas putida, S. enterica, Serratia marcescens, Shigella sonnei, S. maltophilia, V. Fluvialis,*	*Shewanella algae, Vibrio ichthyoenteri, Vibrio scophthalmi,*
qnrB	*C. freundii, C. koseri, E. aerogenes, E. cloacae, E. gergoviae, E. coli, K. ornithinolytica, K. oxytoca, K. pneumoniae, S. enterica, S. marcescens, Shigella spp*	*Citrobacter braakii, Citrobacter freundii, Citrobacter youngae, Citrobacter werkmanii, Serratia marcescens, Enterobacter mori, Serratia proteamaculans, Serratia odorifera, Serratia plymuthica, Serratia sp.*
qnrS	*C. freundii, C. koseri, E. aerogenes, E. cloacae, E. coli, K. oxytoca, K. pneumoniae, Morganella morganii, P. mirabilis, S. enterica, S. typhi, Shigellaa boydii, S. flexneri, S. dysenteriae*	*Vibrionales bacterium, Vibrio cyclitrophicus, Vibrio sp.Vibrio splendidus*
qnrC	*P. mirabilis*	*Vibrio orientalis, Glaciecola arctica, Glaciecola agarilytica, Photobacterium profundum, Aliivibrio salmonicida*
qnrD	*E. coli, C. freundii, K. pneumoniae, M. morganii, P. mirabilis, P. vulgaris, P. rettgeri, P. aeruginosa, S. enterica*	*Dickeya dadantii, Stenotrophomonas maltophilia, Providencia alcalifaciens*
qnrVC	*A. hydrophila, A. punctata, Aeromonas spp., Pseudomonas spp., V. cholera*	*Vibrio cholerae, Vibrio orientalis*
mfpA		*M. Tuberculosis, M. Avium, M. Marinum, M. Smegmatis, M. phlei, M. tuscia, R.opacus, R. imtechensis, R. jostii.*
